# Application of SNP in Genetic Sex Identification and Effect of Estradiol on Gene Expression of Sex-Related Genes in *Strongylocentrotus intermedius*

**DOI:** 10.3389/fendo.2021.756530

**Published:** 2021-11-11

**Authors:** Ya-Lun Han, Zhi-Hui Sun, Shuai Chang, Bin Wen, Jian Song, Ran-Tao Zuo, Ya-Qing Chang

**Affiliations:** Key Laboratory of Mariculture & Stock Enhancement in North China Sea, Ministry of Agriculture and Rural Affairs, Dalian Ocean University, Dalian, China

**Keywords:** SNP, sea urchin, estradiol, 2b-RAD, sex identification

## Abstract

Sea urchin (*Strongylocentrotus intermedius*) is an economically important mariculture species in Asia, and its gonads are the only edible part. The efficiency of genetic breeding in sea urchins is hampered due to the inability to distinguish gender by appearance. In this study, we first identified a sex-associated single nucleotide polymorphism (SNP) by combining type IIB endonuclease restriction site-associated DNA sequencing (2b-RAD-seq) and genome survey. Importantly, this SNP is located within *spata4*, a gene specifically expressed in male. Knocking down of *spata4* by RNA interference (RNAi) in male individuals led to the downregulation of other conserved testis differentiation-related genes and germ cell marker genes. We also revealed that sex ratio in this validated culture population of *S. intermedius* is not 1:1. Moreover, after a 58-day feeding experiment with estradiol, the expression levels of several conserved genes that are related to testis differentiation, ovary differentiation, and estrogen metabolism were dynamically changed. Taken together, our results will contribute toward improving breeding efficiency, developing sex-controlled breeding, and providing a solid base for understanding sex determination mechanisms in sea urchins.

## Introduction

Sea urchin (*Strongylocentrotus intermedius*) belongs to Echinodermata and is an important commercial species mainly distributed in several Asian countries, such as China, Korea, and Japan (1). It is well known that gonads of sea urchins are rich in unsaturated fatty acids and are the only edible parts for people (2). It has been reported that gender is one of the strongest influencing factors on the commercial value of sea urchin; for example, increasing rates of body size and gonadal growth in male sea urchins have advantages than females (3). In addition, the immunocompetence of female *Paracentrotus lividus* urchins might be superior to that of males (4), and the contents of free amino acids and lipid concentrations are different based on gender. As previously described, the gonads of sea urchins are classified into four stages, namely, intergametogenesis and nutritive phagocyte (NP) phagocytosis (stage 1), pregametogenesis and NP renewal (stage 2), gametogenesis and NP utilization (stage 3), and the end of gametogenesis, NP exhaustion, and spawning (stage 4) (5). Recently, three female-specific markers were identified and could be used to identify the genetic sex of *Mesocentrotus nudus* (6). In addition, the number of genes and micro-RNAs associated with sex determination and sex differentiation have been identified by RNA sequencing (7, 8). However, no literature about sex control was reported, and a large part about sex determination and sex differentiation in sea urchin is still unknown.

With the development of next-generation sequencing (NGS) technologies, restriction site-associated DNA sequencing (RAD-seq) was developed to identify sex-specific markers and discover the mechanisms of sex determination in various species (9–11). In *Mastacembelus armatus*, two male-specific DNA fragments and two single nucleotide polymorphisms (SNPs) were identified by RAD-seq, and the *Tcl* gene was regarded as a candidate sex determination gene (12). In crab species, sex-specific SNPs were identified *via* RAD-seq, and a WZ/ZZ sex determination system was suggested based on male homogamety and female heterogamety in those sex-specific SNP loci (13). Sea cucumber (*Apostichopus japonicus*) also belongs to Echinodermata, a male-specific DNA fragment was verified, and a rapid genetic identifying method was developed by sex-specific primer design coupled with PCR amplification (14). Therefore, sex-specific molecular markers obtained by RAD-seq will be helpful in revealing the genetic basis of sex determination and improving breeding efficiency because genetic sex could be identified at early developmental stages.

Vertebrate steroid hormones play important roles in regulating sex differentiation and maintaining secondary sexual characteristics, especially sex steroid hormones including estradiol-17β (E2), testosterone, and progesterone (15, 16). In several aquatic vertebrates, the hormonal-induced sex reversal technique had been widely used to achieve monosex culture; thus, the quality, output, and economic value had been dramatically increased (17, 18). For example, XY physiological female *yellow catfish* was obtained by E2 treatment, then YY super-males will be obtained by gynogenesis of XY physiological female fish, and finally all-male yellow catfish will be obtained by crossing YY super-males with normal XX males (19). What is more, the YY super-males, XY males, and XX females can be identified by Y chromosome-linked and X chromosome-linked markers (20). It has been reported that vertebrate-type steroids can be measured in some invertebrates, such as in *Scylla serrata* (21) and *Chlamys farreri* (22). However, it is still unclear whether those detected hormones are endogenously synthesized or picked up from the environment (23, 24). For example, mollusks can readily absorb vertebrate steroids from the environment and retain them for several weeks (25); thus, whether mollusks contain endogenously synthesized steroids is highly controversial (26). Moreover, steroid androgen exposure has a significant influence on vitellogenin production, ovarian development, and gene expression in some invertebrates, such as *Portunus trituberculatus* (27) and *Saccostrea glomerata* (28). In sea urchins, treatment with estradiol dipropionate will change the rate of protein synthesis in oocytes, increase gonad development, accelerate vitellogenesis, and induce maturation of the germ cells (29). Besides, feeding E2 will increase the rate of ovarian growth but inhibited oocyte growth in *Lytechinus variegatus* (30). However, it is still a mystery whether vertebrate-type steroids can be measured in *S. intermedius* and whether they have potential effects on gonadal development and gene expression patterns related to sex differentiation.

Spermatogenesis associated 4 gene (*spata4*) was first identified in human testes and involved in cryptorchidism development (31). Subsequently, the *spata4* gene had been cloned from diverse organisms. In Mammalia, *SPATA4* is specifically expressed in the testis and may play a significant role in maintaining spermatogenesis ability during adolescence (31, 32). In chicken, the *spata4* gene is also specifically expressed in the testis, and along with development progress of the testis, its expression level is gradually upregulated (33). In zebrafish and rainbow trout (*Oncorhynchus mykiss*), the *spata4* gene is expressed specifically in the testis and slightly in the ovary (34, 35). Moreover, the potential function of *spata4* had been investigated by *in vitro* cell experiment, and it was revealed that regulatory factor X1 (RFX1) could bind the *spata4* promoter, regulating its transcriptional activity and endogenous expression, and further regulate the proliferation of Sertoli cells (36). To date, no literature about *spata4* was reported in sea urchin.

In the present study, 2b-RAD-seq technology was employed to identify sex‐specific tags and SNP loci of sea urchin (*S. intermedius*). Subsequently, we investigated the 2b-RAD markers by PCR in 100 individuals, and a sex-related SNP locus had been identified. Based on the annotation of the sex-related SNP, a male specifically expressed gene *spata4* had been characterized. Then, the function implication of *spata4* in testis development had been investigated by means of RNAi. Furthermore, we measured vertebrate-type steroids in *S. intermedius* and detected the effect of dietary administration of estradiol on testis development and sex-related gene expression in male individuals. Besides the practical implications for improving breeding efficiency, these findings also provide a molecular basis for understanding sex determination mechanisms in sea urchin.

## Materials and Methods

### Sample Preparation and Phenotypic Sex Identification

Twenty sea urchin individuals (10 males and 10 females), randomly collected from the Key Laboratory of Mariculture & Stock Enhancement in North China Sea, Ministry of Agriculture and Rural Affairs, Dalian Ocean University, were used to identify the potential sex-specific markers by 2b-RAD-seq. In addition, 100 aquaculture individuals of *S. intermedius* were purchased from markets in Dalian Haibao Fishery Limited Company and used to confirm the sex-specific markers. The tube feet tissues of each sea urchin were removed and stored in absolute ethanol at −20°C for genomic DNA extraction. Genomic DNA was extracted using the protocols of a marine animal tissue genomic DNA extraction kit (Tiangen, DP324).

To determine the phenotypic sex of individuals, the corresponding gonadal tissues were removed and fixed in 4% paraformaldehyde (PFA) at 4°C overnight, and then the gonadal tissues were washed three times with phosphate buffered solution (PBS) at room temperature. Subsequently, the gonadal tissues were embedded in optimal cutting temperature (O.C.T) compound and frozen sections (5 µm) were cut. After stained with hematoxylin/eosin, the gonadal structure was observed in Leica DM4B microscope to determine the phenotypic sex of each sea urchin.

### Library Construction and 2b‐RAD Sequencing

The 2b-RAD libraries were constructed at Qingdao OE Biotech Co., Ltd. (Qingdao, China) according to a previously report (37). In brief, 1 U *Bsa*XI restriction enzyme was used to digest 200 ng genomic DNA from each individual (10 females and 10 males). The digestion products were ligated to adapter 1 and adapter 2. Then, a set of four primers that introduce individual specific barcodes and sequencing primers was used to amplify the ligation products by PCR. The PCR products were separated in 8% polyacrylamide gels by electrophoresis. After purification, each product was digested with *Sap*I restriction enzyme and purified using streptavidin-coated magnetic beads, and 200 U T4 DNA ligase was added to the supernatant. After purification of the ligation products, barcodes were introduced by PCR with barcode-bearing primers. Then, the PCR products were purified using a MinElute PCR Purification Kit and pooled for sequencing using the Illumina NovaSeq 6000 PE150PE platform.

### Data Filtering, Survey Genome, and Screening of Candidate Sex-Specific Markers

The raw reads were merged by Pear software (version 0.9.6) (38), and then the adaptor sequences of the merged reads were trimmed. The terminal 3-bp positions were also removed from each read. Then, low-quality reads that contain ambiguous bases (N) exceeding 8% or without restriction sites were excluded from the raw data and removed from each output read, and clean reads were obtained. The genomic information of *S. intermedius* is currently unavailable. Also, the 2b-RAD tags we obtained were too short to design PCR primers. One male and one female individual were thus selected to perform short-read *de novo* sequencing. For the genome survey, DNA was extracted from one female and one male *S. intermedius* for library construction by using TruSeq DNA LT Sample Prep kit. Briefly, the genomic DNA was sheared into fragments with length ~350 bp using S220 Focused-ultrasonicators (Covaris, USA). Adapters were ligated onto the 3′ end of the sheared fragments. After PCR amplification and purification, the final libraries were sequenced on the Illumina NovaSeq platform (Illumina Inc., USA) and 150 bp paired-end reads were generated. The above 150-bp paired-end reads were clustered to build reference sequences for further locus genotyping using ustacks software version 1.34 (39). To identify candidate sex-specific markers, high-quality reads were aligned to the genome reference using SOAP2 (version 2.21) with the following parameters: *r* = 0, *M* = 4, *v* = 2 (40). SNP loci were developed by RAD typing program with default parameter. Finally, candidate sex-specific markers for each individual were generated for further analyses.

### Validation of Sex-Specific Markers

PCR amplification was used to validate each candidate sex-specific tag and SNP locus. Firstly, specific primers of candidate sex-specific markers were designed using online software (http://biotools.nubic.northwestern.edu/OligoCalc.html) according to the flank sequences (**Supplementary Table S1**). Then, the PCR reactions were carried out with a total volume of 20 μl, containing 50 ng template DNA, 10 µl of 2× Taq buffer (Kang Wei, China), and 0.5 μl of each primer (10 μM), and sterile water was added to the final volume. The PCR programs were as follows: predenaturation at 94°C for 2 min, then 30 cycles of 94°C for 30 s, annealing temperature of primer pair for 30 s, followed by 72°C for 60 s, and a final extension of 10 min at 72°C. The PCR products were separated *via* 1% agarose gel electrophoresis, and the expected PCR fragments were directly sequenced in both directions by Tsingke Biotechnology (Tianjin, China).

### RNA Extraction, Rapid Amplification of cDNA End, and Real-Time Quantitative PCR

Total RNA was extracted from different tissues, including the ovary, testis, tube feet, and intestines, using the SV Total RNA Isolation System (Promega Z3100). Total RNA (1 µg) of the testis was used to establish a cDNA library using the SMARTer™ Rapid Amplification of cDNA End (RACE) cDNA Amplification Kit (Clontech 634923) according to the recommendations and protocols of the manufacturer. Meanwhile, the first-strand cDNAs of each sample were synthesized according to the protocols of RevertAid First Strand cDNA Synthesis Kit (Fermentas). Real-time quantitative PCR (RT-qPCR) was performed on the LightCycler^®^96 Instrument (Roche) according to a previous description (41), and *18S ribosomal RNA* (*18S rRNA*) was used as the internal control. The relative expression of the target genes was calculated with the 2^−ΔΔCt^ method. For statistical analysis, one-way ANOVA was calculated with SPSS software and a probability (*p*) of <0.05 was considered statistically significant. All primers used were designed by online software http://biotools.nubic.northwestern.edu/OligoCalc.html (**Supplementary Table S1**).

### RNA Interference

The gene-specific dsRNA of *spata4* was designed by https://www.dkfz.de/signaling/e-rnai3/ and synthesized by the T7 RiboMAX™ Express RNAi System (Promega) according to the protocol. Firstly, the genetic sex of sea urchins was identified by PCR reaction and Sanger sequencing depended on the sex-specific SNP marker. Then, a total of nine male *S. intermedius* with a mean shell diameter of 52.5 ± 2.10 mm was selected to inject dsRNA. Meanwhile, the testis development of selected *S. intermedius* was during gametogenesis and NP utilization (stage 3), and this is a critical stage in spermatogenesis. Before injection, three sea urchins were randomly removed and their gonads were surgically sampled as control samples. Then, *spata4*-specific dsRNA was heated to 95°C for 3min and cooled to room temperature (25–30°C). Subsequently, sea urchin was injected with 100 µl PBS containing 100 µg dsRNA according to a previous report (5). On day 3 after the first injection, three sea urchins were randomly removed and their gonads were surgically sampled. Then, a second injection was performed on the remaining sea urchins. On day 7 after the first injection, the gonadal tissues of remaining three sea urchins were surgically sampled. Histological examination of the gonads was carried out according to the method above. RT-qPCR was performed to investigate the knockdown effect and the dynamic expression change of other conserved sex-related genes.

### Enzyme-Linked Immunosorbent Assay and Dietary Administration of Estradiol

*Strongylocentrotus intermedius* used for this experiment were purchased from markets in Dalian Haibao Fishery Limited Company, and after being acclimatized to laboratory conditions for 2 weeks, E2 concentration in the gonadal tissues was detected *via* a commercial enzyme-linked immunosorbent assay (ELISA) kit (Fish estradiol ELISA kit, Mlbio) according to the protocol of the manufacturer. A total of 54 sea urchins individuals with a mean diameter of 31.3 ± 0.43 mm were randomly divided into three groups and cultured in separate seawater tanks at 15°C–17°C. In addition, we made little plastic frames and put them in the seawater tanks, and each sea urchin was cultured and fed on the little plastic frame independently. Among the three groups, two experimental groups had diets with different estradiol contents, one group was fed with food mixed with 50 mg/kg E2 and another group was fed with food mixed with 100 mg/kg E2, while the control group was fed without E2. E2 was purchased from Sigma-Aldrich (E8875-250). Feeding experiments lasted 58 days, and sea urchins were fed with dietary supplementation of E2 every day. On days 3, the excrement of sea urchins was collected to detect E2 level. In addition, on days 15, 30, and 58, six individuals were randomly removed from each group, and their gonads were sampled to analyze the E2 level and for histological examination.

### Transcriptome Sequencing, Assembly, Annotation, and Discovery of Differentially Expressed Unigenes

On day 58 after the feeding experiments, 12 gonadal samples from the control group and those fed with 100 mg/kg E2 were selected for RNA extraction and transcriptome sequencing. For transcriptome sequencing, every three gonadal samples from the same group were mixed as one sequencing sample and each group had two sequencing samples. The transcriptome sequencing was performed on the Illumina sequencing platform (HiSeqTM 2500 or Illumina HiSeq X Ten) by Qingdao OE Biotech Co., Ltd. (Qingdao, China) according to a previous report (42). The clean reads were obtained after being trimmed off of reads with ploy-N and of low quality. To assemble the clean reads, *de novo* assemblies were carried out with Trinity (version: trinityrnaseq_r20131110) software. Subsequently, the assembled unigenes were used to perform the BLAST program on the databases of NCBI RefSeq (NR), Swiss-Prot, and KOG protein databases. In order to identify the differentially expressed genes (DEGs), FPKM and read count value of each annotation unigene was calculated using bowtie2 and eXpress, and DEGs were identified using the DESeq functions estimateSizeFactors and nbinomTest with the following parameters: *p* < 0.05 and fold change >2 or fold change <0.5. Then, DEGs were assigned to different Gene Ontology (GO) terms using the Blast2GO software. Meanwhile, DEGs were blasted against the Kyoto Encyclopedia of Genes and Genomes (KEGG) database by using the automated assignment server (KAAS, http://www.genome.jp/kaas-bin/kaas_main) to identify the biological pathways of these unigenes.

### Multiple Sequence Alignment and Phylogenetic Analyses

The amino-acid sequence was predicted by using the DNAMAN software. Multiple sequence alignment was performed using ClustalW. The phylogenetic trees were constructed using the neighbor-joining method in MEGA version 7.0, with 1,000 replicates of bootstrap analysis. The sequences used in this study were downloaded from the NCBI database (**Supplementary Table S2**).

## Results

### 2b-RAD-Seq Data Analysis

In total, the 2b-RAD-seq produced 140,265,655 raw reads (GenBank: PRJNA761225), and we obtained 131,644,844 clean reads after filtering low-quality reads. In order to cluster and assemble these clean reads, two female and two male samples were selected as references based on read quality and quantity. Following alignment with reference samples, we obtained 979,411 putative tags with an average depth of 33.75× from all 10 female and 10 male samples (**Table 1**). In addition, 43,922 putative SNP loci were screened by alignment analysis.

**Table 1:** Summary of 2b-RAD data obtained from 10 male and 10 female *Strongylocentrotus intermedius*.

Name	Gender	Raw Reads	Clean Reads	Tags	Sequencing Depth
F1	Female	5,983,929	5,623,939	48,632	29.58
F2	Female	5,983,929	5,629,286	48,844	29.01
F3	Female	5,983,929	5,638,825	47,767	28.75
F4	Female	5,983,929	5,620,946	47,849	29.00
F5	Female	5,983,929	5,544,541	48,898	28.91
F6	Female	7,389,608	6,925,516	49,433	36.62
F7	Female	7,389,608	6,943,441	49,675	35.06
F8	Female	7,389,608	6,946,886	48,765	35.16
F9	Female	7,389,608	6,944,941	49,016	35.06
F10	Female	7,389,608	6,801,838	49,235	35.21
M1	Male	8,065,553	7,626,951	49,378	40.47
M2	Male	8,065,553	7,662,999	50,167	37.96
M3	Male	8,065,553	7,646,038	49,089	38.86
M4	Male	8,065,553	7,648,063	49,250	38.44
M5	Male	8,065,553	7,553,171	49,824	38.55
M6	Male	6,614,041	6,182,752	48,796	32.89
M7	Male	6,614,041	6,189,270	49,302	31.31
M8	Male	6,614,041	6,206,661	47,963	31.40
M9	Male	6,614,041	6,192,075	48,335	31.45
M10	Male	6,614,041	6,116,705	49,193	31.37
Average	/	7,013,282.75	6,582,242.2	48,970.55	33.753
Total	/	140,265,655	131,644,844	979,411	675.06

### Genome Survey and Validation of Candidate Sex-Specific Tags and SNPs

A total of 102.37G clean reads with a GC content of 37.16% and 99.94G clean reads with a GC content of 36.75% were acquired from the female and male samples, respectively (GenBank: PRJNA762987). The corresponding draft genomes were then assembled for both female and male. In order to identify sex-specific tag candidates, the 2b-RAD tags of each sample were aligned with both the female and male genome survey. We obtained 38 male-specific candidates that present exclusively in 10 male samples and 13 female-specific tags that only exist in 10 female samples (**Supplementary Table S3, Additional Fasta File 1**). Since a smaller *p*-value of SNP means a higher correlation with sex, the top 10 sex‐specific SNP loci were screened with the lowest *p*-values for further analysis. Notably, the females appeared to be heterozygous, whereas the males seemed to be homozygous for all 10 sex-specific SNP loci candidates in all samples we analyzed (**Supplementary Table S4, Additional Fasta File 2**), indicating that *S. intermedius* may have an ZW/ZZ sex determination system.

**Figure 1:**
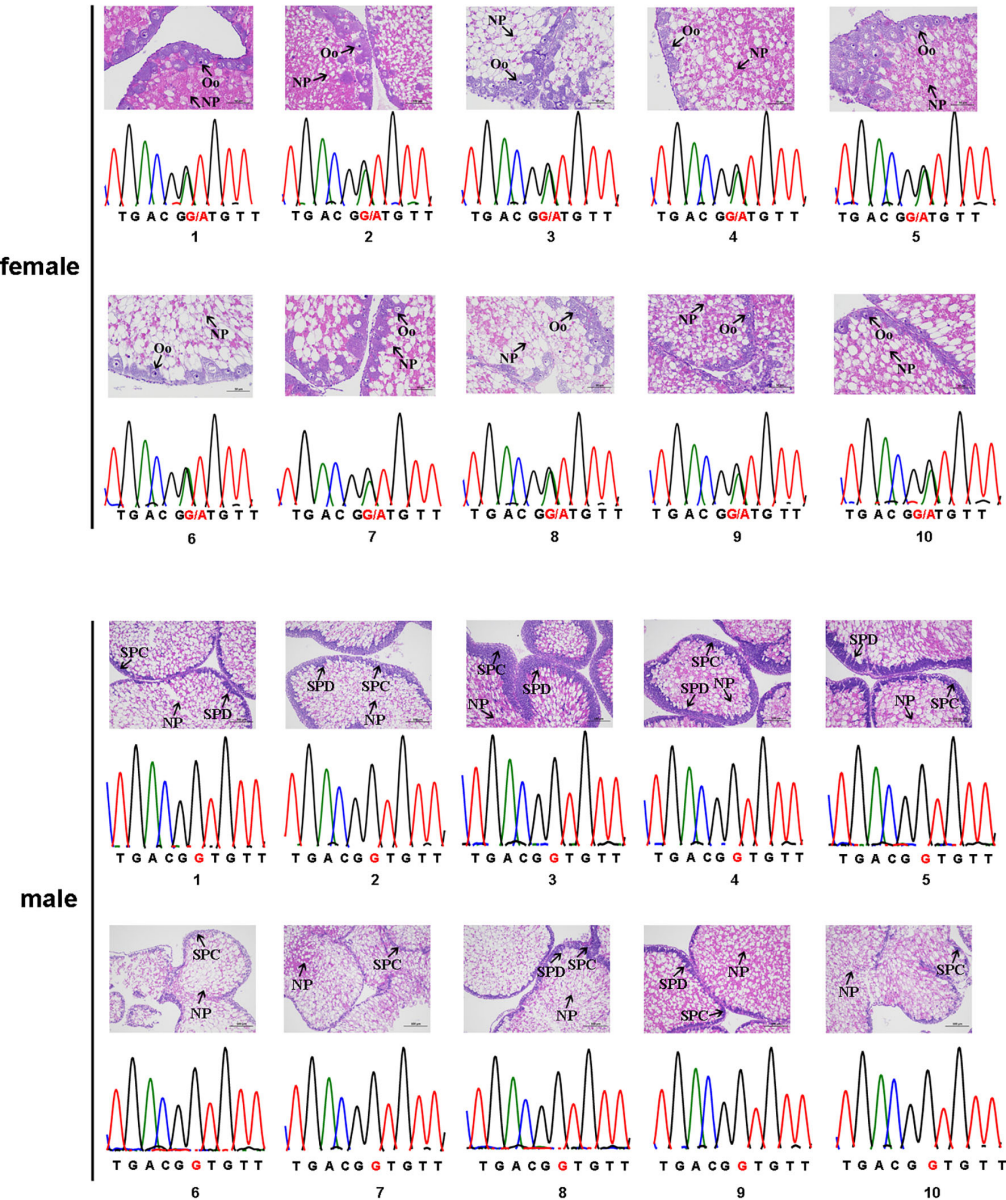
The sex-associated SNP of the *Strongylocentrotus intermedius*. The female genotype is G/A heterozygous and the male genotype is G/G homozygous. NP, nutritive phagocytes; SPC, spermatocyte; Oo, oocyte; SPD, spermatid.

Among the 51 candidate sex-specific tags identified initially, six were too short to be analyzed and were thus excluded. These were female-tag 2, female-tag 5, male-tag 6, male-tag 12, male-tag 13, and male-tag 16. To confirm the authenticity of the remaining 45 tags, we designed primers based on the flanking sequences of these tags and performed PCR verification in 10 female and 10 male sea urchins. Unfortunately, we obtained the same PCR products from all 45 tags in both male and female sea urchins. Sanger sequencing analysis of the PCR products suggested that no difference was observed between male and female. Therefore, all these candidate sex-specific tag candidates were excluded from further study. Next, the same method was used to assess the authenticity of the 10 candidate SNP loci. PCR amplification suggested that nine of them did not show any difference between male and female. Hence, these nine sex-related SNP loci candidates were also excluded for any further analysis. Nonetheless, Sanger sequencing of the PCR products indeed confirmed one candidate sex-specific SNP locus (ref128486). Interestingly, all male individuals we analyzed displayed an apparently homozygous type (G/G), whereas the female individuals showed a G/A heterozygous type (**Figure 1**). Together, these data suggest that this sex-specific SNP may be used to identify the genetic sex of *S. intermedius*.

### Genetic Sex Validation of *Strongylocentrotus intermedius* in Breeding Populations

To assess whether the above sex-specific SNP locus can be used to characterize the phenotypic sex of sea urchin, we ran PCR reactions with the same primers in 100 sea urchins. Prior to PCR amplification, gonadal histology analyses were performed to determine the phenotypic sex of each sea urchin and 65 females and 35 males were identified (**Supplementary Figure S1**). Among all 65 phenotypic female individuals, 56 (86.15%) exhibited the G/A heterozygous genotype (the dominant female genotype) and 9 (16.07%) exhibited the A/A homozygous genotype at the SNP locus. For the rest of the phenotypic male individuals, 33 (94.29%) exhibited the G/G homozygous genotype (the dominant male genotype) and 2 (5.71%) exhibited the G/A heterozygous genotype at the SNP locus (**Table 2**). Despite the fact that high consistency (89%) was obtained rapidly from the PCR amplification, the PCR products from 11 individuals (11%) were inconsistent with their phonotypic sex. Importantly, the segregation ratio of female to male was 65:35 (1.86:1) in this culture population. The above data indicate that sex reversal is possible in *S. intermedius*.

**Table 2:** SNP genotype and phenotypic sex of the *Strongylocentrotus intermedius*.

SNP genotype	Phenotypic male	Phenotypic female
G/A	2	56
G/G	33	0
A/A	0	9

### Annotation of Sex-Associated SNP Sequence

**Figure 2:**
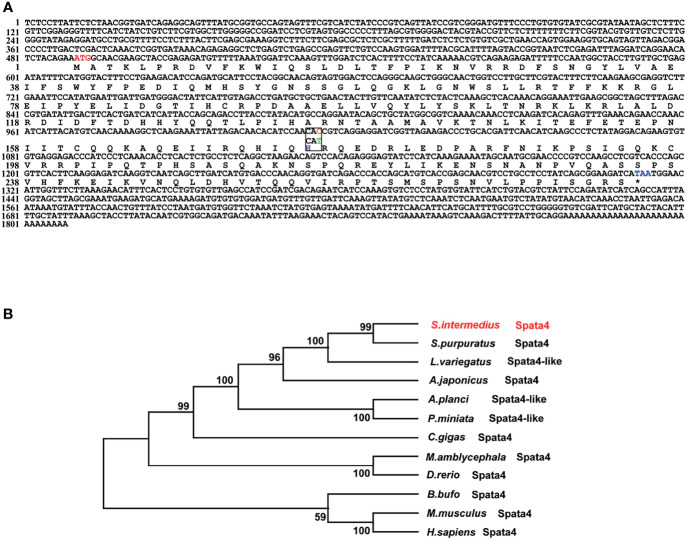
Sequence analysis of *spata4* in *S. intermedius*. **(A)** Coding sequence and deduced amino acid sequences of *spata4* in *S. intermedius.* **(B)** Phylogenetic tree of Spata4 proteins.

**Figure 3:**
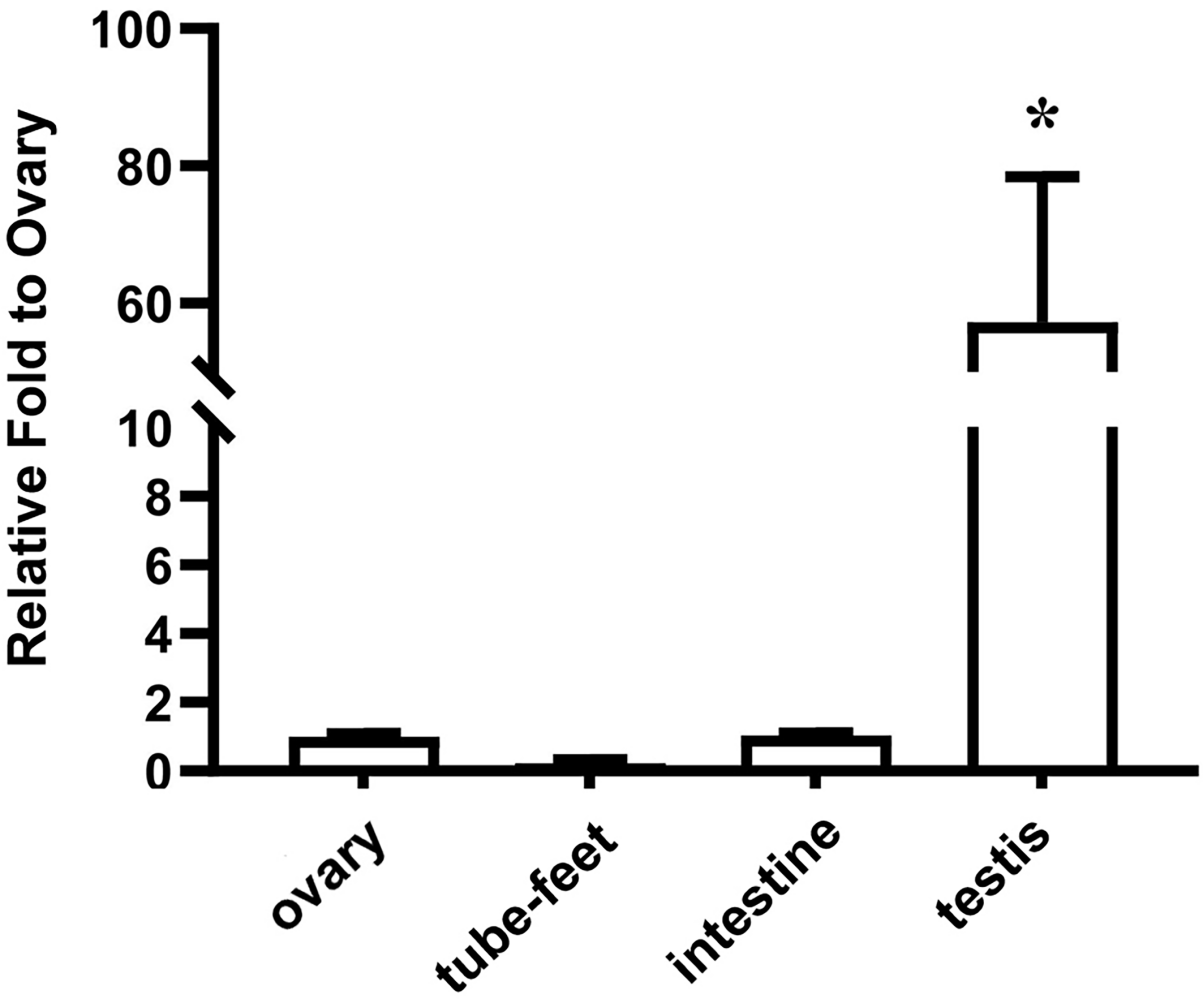
Expression pattern of *spata4* mRNA in *S. intermedius. 18S* was used as the control. Each bar represents mean ± standard deviation (SD) (*n* = 3). One-way ANOVA was used to determine statistical analysis. Asterisks (*) indicate significant differences (*p* ≤ 0.05).

The sex-associated SNP locus is located on scaffold ref128486. Ref128486 is 6,149 bp in length. To search for homologous genes on the scaffold, we performed a BLAST program on NCBI databases. Interestingly, a spermatogenesis-associated (SPATA) family gene (*spata4*) was identified, and it has been reported that SPATA family genes play important roles in spermatogenesis, sperm maturation, and fertilization (43). The *spata4* cDNA is 1,808 bp in length and contains an 825-bp open reading frame (ORF) that encodes a protein of 274 amino acids, a 489-bp 5′UTR, and a 494-bp 3′UTR. Because of the presence of an SNP locus, the 175th codon of the *spata4* gene is CAC in males but CAU in females. However, codons of CAC and CAU are both coding for histidine (**Figure 2A**). Multiple protein sequence alignment analysis revealed that the Spata4 protein exhibited a high level of identity with other homologs, especially with the echinoderm, including *Strongylocentrotus purpuratus* (99.27%), *L. variegatus* (86.18%), and *Acanthaster planci* (63.16%; **Supplementary Figure S2**). Phylogenetic analysis showed that the topology of clades is basically consistent with the known taxonomic relationship among these analyzed species (**Figure 2B**). In addition, the mRNA expression pattern of *spata4* in *S. intermedius* was detected by RT-qPCR in different adult tissues, including the testis, ovary, tube feet, and intestines. The results suggested that *spata4* is expressed predominantly in the testis. By contrast, only a few of the *spata4* transcripts can be detected in the ovary and intestine (**Figure 3**).

### Knockdown of *spata4* by RNAi in *Strongylocentrotus intermedius*

**Figure 4:**
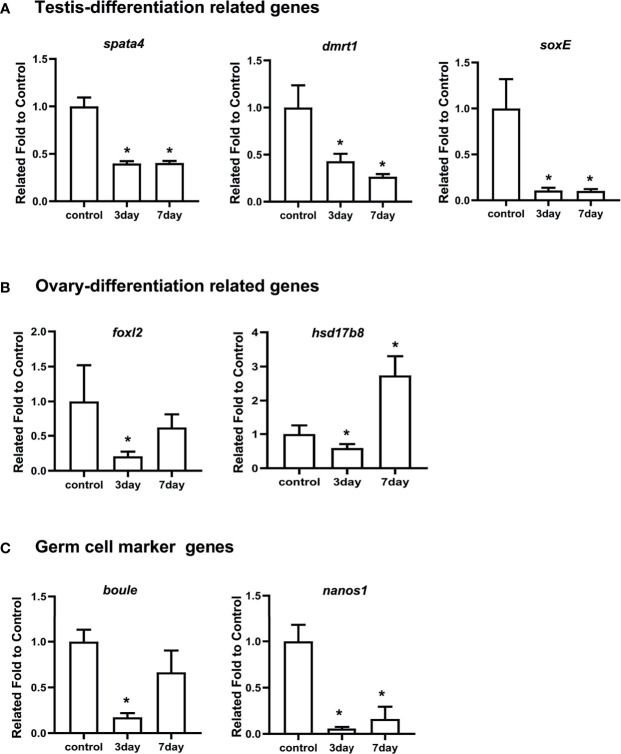
RT-qPCR measured the mRNA levels of *spata4* and other sex-related genes after RNAi. **(A)** The expression of testis differentiation genes, including *spata4*, *dmrt1*, and *soxE*. **(B)** The expression of ovary differentiation genes, including *foxl2* and *hsd17b8*. **(C)** The expression of germ cell marker genes, including *boule* and *nanos1*. *18S* was used as the control. The gene expression in the control group was set to 1. Each bar represents mean ± standard deviation (SD) from three individuals. Independent-sample *t*-test was used to perform statistical analysis. Asterisks (*) indicate significant differences (*p* ≤ 0.05) between knockdown and control.

Considering that *spata4* is expressed predominantly in the testis (**Figure 3**), we further explored its function in spermatogenesis by RNAi, a strategy that has been successfully utilized to study sex-related gene function in sea urchin (5). The genetic sex of nine sea urchins were first identified by PCR amplification and the corresponding products were sequenced. Gonadal tissues from three male sea urchins were removed and sampled as a control group before injection. The specific *spata4*-targeting double-stranded RNA (dsRNA) was then injected into adult *S. intermedius*. At 3 and 7 days post injection (dpi), gonadal samples were collected to perform histological analysis and to detect the *spata4* mRNA expression with RT-qPCR. We observed that, compared with the control groups, the expression levels of *spata4* in the testis were significantly decreased (about 60%) in knockdown samples (**Figure 4A**). Moreover, the results of histological examination showed that no obvious apoptotic cells were observed in the dsRNA-treated sea urchins (**Supplementary Figure S3**).

We also measured the expression levels of other well-studied genes involved in testis differentiation, ovary differentiation, and germ cell development. As shown in **Figure 4A**, the expression of *dmrt1* and *soxE* in the testis differentiation signaling pathway significantly decreased in *spata4*-knockdown samples compared with the control groups at 3 and 7 dpi. Interestingly, the transcripts of *foxl2* and *hsd17b8* were similarly suppressed significantly in *spata4*-knockdown samples at 3 dpi, but the *hsd17b8* expression increased about 2.74-fold in the knockdown samples at 7 dpi (**Figure 4B**). In addition, the germ cell marker genes, including *boule* and *nanos1* expression, were also significantly decreased after injection in knockdown samples (**Figure 4C**).

### Dietary Administration of Estradiol in Male *Strongylocentrotus intermedius*

**Figure 5:**
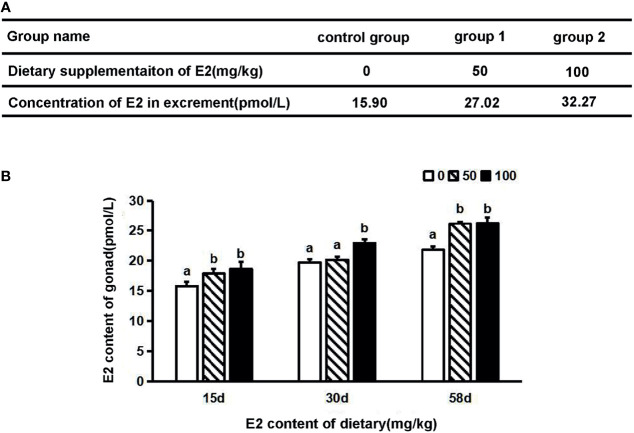
The concentration of estradiol levels in *S. intermedius*. **(A)** Concentration of estradiol in excrement after 3 days of dietary supplementation of E2. **(B)** Concentration of estradiol in testis on different days after dietary supplementation of E2 (*n* = 6). E2, estradiol; d, day. One-way ANOVA was used to determine statistical analysis. Different letters indicate significant differences (*p* *≤* 0.05).

Invertebrates show a high degree of variability in sex, and phenotypic sex may be affected by environmental factors, such as sex hormone, temperature, and stocking density (44). Because the genetic sex of 11 individuals identified by PCR amplification was inconsistent with their phonotypic sex and the sex ratio of females to males was not 1:1, we speculated that sex reversal is possible in *S. intermedius*. To test this hypothesis, we first determined the level of E2 in the gonads of *S. intermedius* by ELISA and found that the concentration of E2 was 17.4 and 13.75 pmol/L in the ovary and testis, respectively. We then characterized male sea urchins with the above SNP and feed them with formulated diet supplemented with 50 or 100 mg/kg E2 (defined as group 1 and group 2, respectively) for 58 days. At 3 days post feeding (dpf), the excrement was collected to detect E2 level. As shown in **Figure 5A**, the E2 concentration increased along with the dietary supplementation of E2. Gonadal tissues were also collected at 15, 30, and 58 dpf to examine the concentration of E2. As shown in **Figure 5B**, the E2 concentrations from sea urchins fed with E2 were comparable with those of the control group during a period of 15 days. On day 30, the concentrations of E2 in group 2 were 1.16 times higher than those of the control group. On day 58, the E2 concentrations from group 1 and group 2 were 1.20- and 1.20-fold against the control group, respectively (**Figure 5B**). These data suggested that *S. intermedius* can absorb estradiol from food and accumulate in the gonads.

### Comparative Transcriptome Analysis of *Strongylocentrotus intermedius* Between E2 Treatment and Wild Type

**Figure 6:**
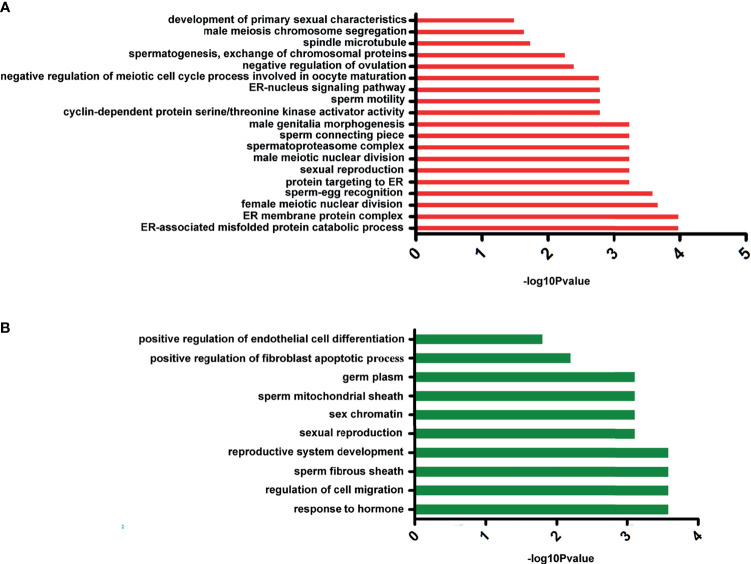
The significant Gene Ontology (GO) annotation of differentially expressed genes. **(A)** The GO cluster of upregulated genes. **(B)** The GO cluster of downregulated genes.

To explore the dynamic expression changes of sex-related genes after E2 treatment, we performed comparative transcriptome analysis to investigate the expression characteristics and differentially expressed genes (DEGs) in the control group and in the 100-mg/kg E2-treated sea urchins (i.e., group 2). In total, 96.13M and 94.57M clean reads were acquired from group 2 and the control group libraries, respectively (GenBank: PRJNA761244). After assembly with *de novo* methods and functional annotation, 67,492 unigenes were identified. The expression levels of unigenes were then compared, and 2,355 DEGs with at least two-fold changes (*p *< 0.05) were identified. Of these DEGs, 1,050 were downregulated and 1,305 were upregulated in the E2 treatment group. In order to categorize the transcripts according to putative function, GO assignment was performed on all DEGs. Not surprisingly, several upregulated genes were enriched significantly in GO terms that are related to estrogen metabolism, spermatogenesis, and oogenesis (**Figure 6A**). These include development of primary sexual characteristics (GO:0045137), sperm-egg recognition (GO:0035036), ER-nucleus signaling pathway (GO:0006984), and sexual reproduction (GO:0019953). By contrast, many downregulated genes were significantly enriched in terms of germ plasm (GO:0060293) and positive regulation of fibroblast apoptotic process (GO:2000271) (**Figure 6B**). Searching the downregulated unigenes in the KOG database showed significant enrichment of unigenes in pathways previously implicated in ovarian steroidogenesis (ko04913), drug metabolism (ko00982), and cytochrome P450 estrogen signaling pathway (ko04915). Besides, the pathway of oocyte meiosis (ko04114) was recognized as a significantly upregulated term. Together, these data suggested that E2 treatment leads to downregulation of testis-biased genes but upregulation of ovary-biased genes and genes associated with estrogen metabolism pathway in *S. intermedius.*

**Figure 7:**
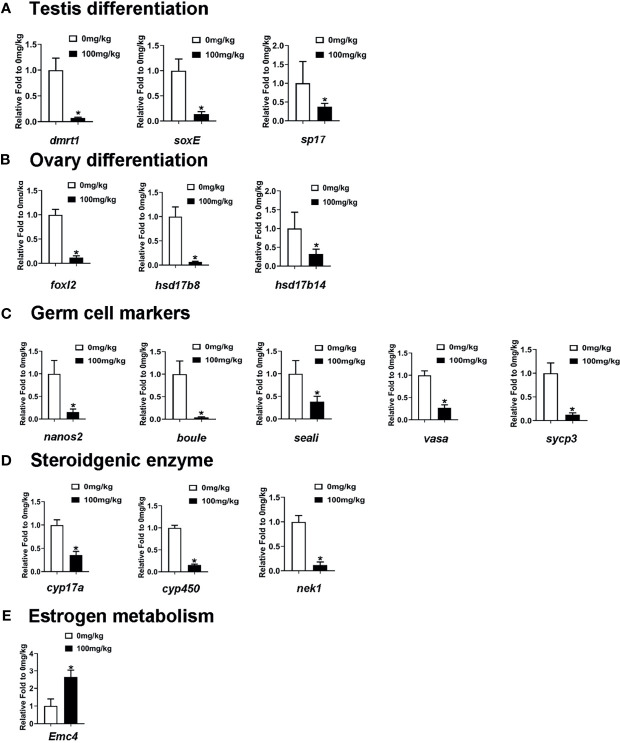
RT-qPCR measured the mRNA levels of sex-related genes and estrogen metabolism-related genes in the testis of E2-treated *S. intermedius*. **(A)** The expression of testis differentiation genes. **(B)** The expression of ovary differentiation genes. **(C)** The expression of germ cell marker gene. **(D)** The expression of steroidgenic enzyme gene. **(E)** The expression of estrogen metabolism gene (Emc4). *18S* was used as the control. Each bar represents mean ± standard deviation (SD) (*n* = 3). One-way ANOVA was used to determine statistical analysis. Asterisks (*) indicate significant differences (*p* ≤ 0.05).

Lastly, we analyzed the dynamic expression patterns of several sex-related markers that were reported to have been involved in gonad development in the testis of E2-treated *S. intermedius* by RT-qPCR. As shown in **Figure 7A**, testis differentiation-associated genes, including *dmrt1*, *soxE*, and *sp17*, were significantly downregulated in the testis samples of E2-treated *S. intermedius*. Curiously, genes needed for ovary differentiation including *hsd17b8*, *foxl2*, and *had17b14* were also downregulated (**Figure 7B**). Notably, E2 treatment led to a significant decrease in germ cell marker genes including *nanos2*, *boule*, *seali*, *sycp3*, and *vasa* in sea urchins (**Figure 7C**). In addition, steroidgenic enzyme genes related to active androgen biosynthesis such as *cyp17a*, *cyp450*, and *nek1* were also downregulated in the testis of E2-treated sea urchins (**Figure 7D**). As expected, genes associated with estrogen metabolism including *ER membrane protein complex subunit 4* (*Emc4*) were significantly upregulated (**Figure 7E**). However, no phenome of sex reversal was observed in the testis of E2-treated *S. intermedius* according to histological analysis (**Supplementary Figure S4**).

## Discussion

Identification of sex-specific markers is fundamental to understand the sex determination mechanism and is crucial for genetic sex identification and sex-controlled breeding (45, 46). Recently, 2b-RAD-seq has been successfully applied to identify sex-specific markers in a great diversity of aquatic organisms (9, 10). RNA interference is an effective genetic tool for studying gene function *in vivo*, especially in animals with low survival rates and long reproductive cycles (5). Currently, a green fluorescent protein (GFP)-derived double-stranded RNA (dsRNA-GFP) is usually used as control in RNAi studies, and no RNAi effect was detected using dsRNA-GFP suggesting target sequence specificity of the RNAi effect (47). However, according to a previous report in *Apis mellifera*, the expression of nearly 1,400 genes was dynamically changed in response to dsRNA-GFP (48). Hence, it is indeed a big challenge to evaluate target sequence specificity of interference effect.

In pufferfish, a female homozygous (C/C) type and a male heterozygous (C/G) SNP in anti-Mullerian hormone receptor type 2 (*amhr2*) were identified, and this SNP site can be used to identify the genotypic sex of pufferfish with high accuracy (49). Notably, the missense SNP that resulted in an amino acid mutation (His/Asp) in *amhr2* affects its kinase activity, and the combination of the two alleles of *amhr2* may determine the sex of *Takifugu rubripes* (50, 51). In the current study, a sex-linked SNP locus was identified in *S. intermedius* s*pata4* gene. The SNP locus is located at the 175th codon of the *spata4* gene, but the CAC➔CAU change only causes a synonymous mutation in the predicted Spata4 protein. Synonymous mutations are thought to be silent for a long time. Recently, more and more studies reveal that synonymous sites do not evolve neutrally, and synonymous mutations can regulate protein conformation and function by affecting mRNA stability (52, 53). However, the effect of synonymous mutations on the expression and function of *spata4* gene remains unclear and should be investigated in the future. In most of the species, *spata4* is specifically expressed in the testis and may play important roles in maintaining spermatogenesis ability and infertility process (31, 43, 54). Herein, we revealed that *spata4* is expressed predominantly in the testis, and only a few of *spata4* transcripts can be detected in the ovary (**Figure 3**). As previously reported, *dmrt1* and *soxE* were demonstrated to play critical roles in testis differentiation regulatory pathway (55, 56). Meanwhile, *boule* and *nanos1* were previously described as germ cell markers and played conserved roles in regulating germ cell development (57, 58). Knocking down *spata4* expression in sea urchins led to the downregulation of *soxE*, *dmrt1*, *boule*, and *nanos1*, indicating a close correlation between *spata4* expression and testis development.

Although estradiol can be detected in the gonadal tissues of *S. intermedius*, we are uncertain whether it is absorbed from the environment or is endogenously synthesized. It has been reported that vertebrate steroids can be absorbed and utilized by invertebrates, especially those living in water, and all reproductively mature fish can release steroids into the environment (23, 25, 59). Sea urchins (*L. variegatus*, *S. purpuratus*, and *S. intermedius*) are also capable of absorbing vertebrate steroids. A notable example is that estradiol-17β exposure can induce protein synthesis in oocytes of *S. intermedius* (29).

It has been suggested that sex determination and sex differentiation of invertebrates may be influenced by environment factors such as temperature, nutrition level, and photoperiod (44). Because the ratio between female and male was not around 1:1 in the current study, we speculated that sex reversal might be the case in *S. intermedius*. After 58 days of dietary administration of estradiol, we did not observe significant sex reversal in male individuals by gonadal histology analysis. However, it should be noted that the expression levels of multiple genes have changed dynamically after estradiol treatment. In particular, many of these genes are known to be involved in testis differentiation, ovary differentiation, and germ cell development. Thus, it is possible that E2 treatment may affect gonadal development in sea urchin.

A vertebrate homologous estrogen receptor (ER) (ADL71443.1) is annotated in transcription database. Nonetheless, its expression level is relatively stable following E2 treatment, implying that the interacting mechanisms between exogenous estrogens and sea urchins may be different from that of vertebrates. In *Marisa cornuarietis*, estradiol-17β treatment has no obvious impact on the expression levels of ER-like or estrogen-related receptor (ERR) gene. This is not surprising because estradiol-17β does not bind to ER-like or ERR (60, 61). Hence, it is attempted to reveal the interactions between estrogen and estrogen receptor in sea urchin.

In conclusion, the current studies identified a sex-related SNP site in *S. intermedius* and revealed that this SNP is located within the *spata4* gene. We confirmed that s*pata4* is expressed predominantly in the testis, and *spata4* may be involved in testis development. In addition, *S. intermedius* can absorb vertebrate estradiol from dietary food, which caused dynamic changes of the sex-related genes. Taken together, our results provide a powerful tool for understanding the sex-determination mechanisms in sea urchins and have important implications for improving the breeding efficiency of sea urchins.

## Data Availability Statement

The names of the repository/repositories and accession number(s) can be found below: https://www.ncbi.nlm.nih.gov/sra/?term=PRJNA761225, https://www.ncbi.nlm.nih.gov/sra/?term=PRJNA762987, https://www.ncbi.nlm.nih.gov/sra/?term=PRJNA761244. You can also get raw datas by searching the accession numbers (PRJNA761225, PRJNA761244, PRJNA762987) in NCBI. The accession number of spata4 sequence is OK078897. This sequence will be accessible after the indicated release date or the article was published.

## Author Contributions

Conceptualization: Z-HS. Software: Y-LH. Validation: Y-LH, SC, and BW. Investigation, Y-LH. Resources: JS and R-TZ. Data curation: Y-LH. Writing—original draft preparation: Z-HS. Writing—review and editing: Y-QC and Z-HS. Funding acquisition: Y-QC and Z-HS. All authors have read and agreed to the published version of the manuscript.

## Funding

This work was funded by the National Key Research and Development Program of China (2018YFD0901603) and the National Natural Science Foundation of China (31802276).

## Conflict of Interest

The authors declare that the research was conducted in the absence of any commercial or financial relationships that could be construed as a potential conflict of interest.

## Publisher’s Note

All claims expressed in this article are solely those of the authors and do not necessarily represent those of their affiliated organizations, or those of the publisher, the editors and the reviewers. Any product that may be evaluated in this article, or claim that may be made by its manufacturer, is not guaranteed or endorsed by the publisher.

## References

[1] Agatsuma Y. Mesocentrotus Nudus. In: Lawrence JM, editor. Developments in Aquaculture and Fisheries Science, vol. 43. Amsterdam, FL: Elsevier Press (2020). p. 627–41. doi: 10.1016/B978-0-12-819570-3.00034-2

[2] Chang YQ Ding J Song J Yang W. Biology Research and Breeding of Sea Cucumber and Sea Urchin. Beijing: Ocean Press (2004).

[3] Gil DG Zaixso HE Tolosano JA. Sex-Specific Differences in Gonopore and Gonadal Growth Trajectories in the Brooding Sea Urchin, *Abatus Cavernosus* (*Spatangoida*). Invertebr Biol (2020) 139(1):e12278. doi: 10.1111/ivb.12278

[4] Arizza V Vazzana M Schillaci D Russo D Giaramita FT Parrinello N. Gender Differences in the Immune System Activities of Sea Urchin *Paracentrotus Lividus* . Comp Biochem Physiol A: Mol Integr Physiol (2013) 164:447–55. doi: 10.1016/j.cbpa.2012.11.021 23220062

[5] Zhang J Han X Wang J Liu BZ Wei JL Zhang WJ. Molecular Cloning and Sexually Dimorphic Expression Analysis of *Nanos2* in the Sea Urchin, *Mesocentrotus Nudus* . Int J Mol Sci (2019) 20(11):2705. doi: 10.3390/ijms20112705 PMC6600436 31159444

[6] Cui ZP Zhang J Sun ZH Liu BZ Zhao C Chang YQ. Identification of Sex-Specific Markers Through 2b-RAD Sequencing in the Sea Urchin (*Mesocentrotus Nudus*). Fron Genet (2021) 12:1433. doi: 10.3389/fgene.2021.717538 PMC8375557 34422019

[7] Wang H Ding J Ding S Chang YQ. Transcriptome Analysis to Characterize the Genes Related to Gonad Growth and Fatty Acid Metabolism in the Sea Urchin *Strongylocentrotus Intermedius* . Genes Genomics (2019) 41:1397–415. doi: 10.1007/s13258-019-00864-0 31485990

[8] Mi X Wei Z Zhou Z Liu X. Identification and Profiling of Sex-Biased microRNAs From Sea Urchin *Strongylocentrotus Nudus* Gonad by Solexa Deep Sequencing. Comp Biochem Physiol Part D: Genomics Proteomics (2014) 10:1–8. doi: 10.1016/j.cbd.2014.01.001 24486540

[9] Zhou Y Wu J Wang Z Li G Mei J Zhou L. Identification of Sex-Specific Markers and Heterogametic XX/XY Sex Determination System by 2b-RAD Sequencing in Redtail Catfish (*Mystus Wyckioides*). Aquac Res (2019) 50:2251–66. doi: 10.1111/are.14106

[10] Zhu C Liu H Cheng L Pan Z Chang G Wu N. Identification of Sex-Specific Sequences Through 2b-RAD Sequencing in *Pseudobagrus Ussuriensis* . Aquaculture (2021) 539:736639. doi: 10.1016/j.aquaculture.2021.736639

[11] Liu H Pang M Yu X Zhou Y Tong J Fu B. Sex-Specific Markers Developed by Next-Generation Sequencing Confirmed an XX/XY Sex Determination System in Bighead Carp (*Hypophthalmichehys Nobilis*) and Silver Carp (*Hypophthalmichthys Molitrix*). DNA Res (2018) 25(3):257–64. doi: 10.1093/dnares/dsy009 PMC6014435 29315393

[12] Xue LZ Guo XF Zhou YL Wang ZW Fan HP Li DP. Screening and Characterization of Sex-Specific Markers by 2b-RAD Sequencing in Zig-Zag Eel (*Mastacembelus Armatus*) With Implication of XY Sex Determination System. Aquaculture (2020) 528:735550. doi: 10.1016/j.aquaculture.2020.735550

[13] Shi X Waiho K Li X Ikhwanuddin M Miao G Lin F. Female-Specific SNP Markers Provide Insights Into a WZ/ZZ Sex Determination System for Mud Crabs Scylla Paramamosain, S. Tranquebarica and *S. Serrata* With a Rapid Method for Genetic Sex Identification. BMC Genomics (2018) 19:981. doi: 10.1186/s12864-018-5380-8 30594128 PMC6311006

[14] Wei JL Cong JJ Sun ZH Song J Zhao C Chang YQ. A Rapid and Reliable Method for Genetic Sex Identification in Sea Cucumber, *Apostichopus Japonicus* . Aquaculture (2021) 543:737021. doi: 10.1016/J.AQUACULTURE.2021.737021

[15] Mei J Gui JF. Genetic Basis and Biotechnological Manipulation of Sexual Dimorphism and Sex Determination in Fish. Sci China Life Sci (2015) 58:124–36. doi: 10.1007/s11427-014-4797-9 25563981

[16] Li XY Gui JF. Diverse and Variable Sex Determination Mechanisms in Vertebrates. Sci China Life Sci (2018) 61(12):1503–14. doi: 10.1007/s11427-018-9415-7 30443862

[17] Liu H Guan B Xu J Hou C Tian H Chen HJ. Genetic Manipulation of Sex Ratio for the Large-Scale Breeding of YY Super-Male and XY All-Male Yellow Catfish (*Pelteobagrus Fulvidraco* (*Richardson*)). Mar Biotechnol (2013) 15:321–8. doi: 10.1007/s10126-012-9487-7 23053056

[18] Ezaz MT Myers JM Powell SF McAndrew BJ Penman DJ. Sex Ratios in the Progeny of Androgenetic and Gynogenetic YY Male Nile Tilapia, *Oreochromis Niloticus* . Aquaculture (2004) 232(1-4):205–14. doi: 10.1016/j.aquaculture.2003.08.001

[19] Liu H Cui S Hou C Xu J Hong XC. YY Supermale Generated Gynogenetically From XY Female in Pelteobagrus Fulvidraco (*Richardson*). Acta Hydrobiol Sinica (2007) 31(5):725. doi: 10.3321/j.issn:1000-3207.2007.05.018

[20] Dan C Mei J Wang D Gui JF. Genetic Differentiation and Efficient Sex-Specific Marker Development of a Pair of Y-And X-Linked Markers in Yellow Catfish. Int J Biol Sci (2013) 9(10):1043. doi: 10.7150/ijbs.7203 24250249 PMC3831117

[21] Warrier SR Tirumalai R Subramoniam T. Occurrence of Vertebrate Steroids, Estradiol 17β and Progesterone in the Reproducing Females of the Mud Crab *Scylla Serrata* . Comp Biochem Phys A: Mol Integr Physiol (2001) 130(2):283–94. doi: 10.1016/S1095-6433(01)00385-3 11544073

[22] Zheng BH An LH Chang H Liu Y Jiang ZQ. Evidence for the Presence of Sex Steroid Hormones in Zhikong Scallop, *Chlamys Farreri* . J Steroid Biochem Mol Biol (2014) 143:199–206. doi: 10.1016/j.jsbmb.2014.03.002 24662324

[23] Scott AP. Do Mollusks Use Vertebrate Sex Steroids as Reproductive Hormones? Part I: Critical Appraisal of the Evidence for the Presence, Biosynthesis and Uptake of Steroids. Steroids (2012) 77:1450–68. doi: 10.1016/j.steroids.2012.08.009 22960651

[24] Scott AP. Is There Any Value in Measuring Vertebrate Steroids in Invertebrates? Gen Comp Endocr (2018) 265:77–82. doi: 10.1016/j.ygcen.2018.04.005 29625121

[25] Schwarz TI Katsiadaki I Maskrey BH Scott AP. Mussels (*Mytilus Spp.*) Display an Ability for Rapid and High Capacity Uptake of the Vertebrate Steroid, Estradiol-17β From Water. J Steroid Biochem Mol Biol (2017) 165:407–20. doi: 10.1016/j.jsbmb.2016.08.007 27568213

[26] Horiguchi T Ohta Y. Advances in Invertebrate (Neuro)Endocrinology. Oakville: Apple Academic Press (2020).

[27] Andrew MN O’Connor WA Dunstan RH MacFarlane GR. Exposure to 17α-Ethynylestradiol Causes Dose and Temporally Dependent Changes in Intersex, Females and Vitellogenin Production in the Sydney Rock Oyster. Ecotoxicology (2010) 19(8):1440–51. doi: 10.1007/s10646-010-0529-5 20700763

[28] Lu Y Liu M Gong J Cheng Y Wu X. Effect of Exogenous Estrogen on the Ovarian Development and Gene Expression in the Female Swimming Crab *Portunus Trituberculatus* (Miers, 1876) (Decapoda: Brachyura: Portunidae). J Crustacean Biol (2018) 38:367–73. doi: 10.1093/jcbiol/ruy013

[29] Varaksina GS Varaksin AA. Effect of Estradiol Dipropionate on Protein Synthesis in Oocytes and Ovary of the Sea Urchin Strongylocentrotus Intermedius at Different Stages of the Reproductive Cycle. Biol Bull Russian Acad Sci (2002) 29:96–500. doi: 10.1023/A:1020422028333 12400385

[30] Wasson K Gower B Watts SA. Responses of Ovaries and Testes of *Lytechinus Variegatus* (Echinodermata: Echinoidea) to Dietary Administration of Estradiol, Progesterone and Testosterone. Mar Biol (2000) 137:245–55. doi: 10.1007/s002270000360

[31] Liu SF He S Liu BW Zhao Y Wang Z. Cloning and Characterization of Testis-Specific Spermatogenesis Associated Gene Homologous to Human SPATA4 in Rat. Biol Pharm Bull (2004) 27:1867–70. doi: 10.1248/bpb.27.1867 15516739

[32] Liu SF Ai C Ge ZQ Liu HL Liu BW He S. Molecular Cloning and Bioinformatic Analysis of SPATA4 Gene. J Biochem Mol Biol (2005) 38:739–47. doi: 10.5483/BMBRep.2005.38.6.739 16336790

[33] Xie MC Ai C Jin XM Liu SF Tao SX Li ZD. Cloning and Characterization of Chicken SPATA4 Gene and Analysis of Its Specific Expression. Mol Cell Biochem (2007) 306(1):79–85. doi: 10.1007/s11010-007-9556-9 17673952

[34] Liu SF Liu BW He S Zhao Y Wang Z. Cloning and Characterization of Zebra Fish SPATA4 Gene and Analysis of Its Gonad Specific Expression. Biochem (Moscow) (2005) 70:638–44. doi: 10.1007/s10541-005-0163-7 16038605

[35] Liu BW Liu SF He S Zhao Y Hu H Wang Z. Cloning and Expression Analysis of Gonadogenesis-Associated Gene SPATA4 From Rainbow Trout (*Oncorhynchus Mykiss*). BMB Rep (2005) 38(2):206–10. doi: 10.5483/bmbrep.2005.38.2.206 15826498

[36] Jiang J Zhang N Shiba H Li L Wang Z. Spermatogenesis Associated 4 Promotes Sertoli Cell Proliferation Modulated Negatively by Regulatory Factor X1. PloS One (2013) 8(10):e75933. doi: 10.1371/journal.pone.0075933 24146794 PMC3795713

[37] Wang S Meyer E McKay JK Matz MV. 2b-RAD: A Simple and Flexible Method for Genome-Wide Genotyping. Nat Methods (2012) 9(8):808–8109. doi: 10.1038/nmeth.2023 22609625

[38] Zhang J Kobert K Flouri T Stamatakis A. PEAR: A Fast and Accurate Illumina Paired-End Read Merger. Bioinformatics (2014) 30(5):614–20. doi: 10.1093/bioinformatics/btt593 PMC3933873 24142950

[39] Catchen JM Amores A Hohenlohe P Cresko W Postlethwait J.H.J.G.G. Stacks: Building and Genotyping Loci De Novo From Short-Read Sequences. G3: Genes Genomes Genet (2011) 1:171–82. doi: 10.1534/g3.111.000240 PMC3276136 22384329

[40] Li R Yu C Li Y Lam TW Yiu SM Kristiansen K. Soap2: An Improved Ultrafast Tool for Short Read Alignment. Bioinformatics (2009) 25:1966–7. doi: 10.1093/bioinformatics/btp336 19497933

[41] Sun ZH Wei JL Cui ZP Han YL Zhang J Song J. Identification and Functional Characterization of *Piwi1* Gene in Sea Cucumber, *Apostichopus Japonicas* . Comp Biochem Physiol Part B: Biochem Mol Biol (2021) 252:110536. doi: 10.1016/j.cbpb.2020.110536 33212209

[42] Sun ZH Zhang J Zhang WJ Chang YQ. Gonadal Transcriptomic Analysis and Identification of Candidate Sex-Related Genes in *Mesocentrotus Nudus* . Gene (2019) 698:72–81. doi: 10.1016/j.gene.2019.02.054 30825598

[43] Sujit KM Singh V Trivedi S Singh K Gupta G Rajender SJA. Increased DNA Methylation in the Spermatogenesis-Associated (SPATA) Genes Correlates With Infertility. Andrology (2020) 8(3):602–9. doi: 10.1111/andr.12742 31838782

[44] Cook JM. Sex Ratios: Concepts and Research Methods. Cambridge: Cambridge University Press (2002)

[45] Arslan T Phelps RPJA. Production of Monosex Male Black Crappie, *Pomoxis Nigromaculatus*, Populations by Multiple Androgen Immersion. Aquaculture (2004) 234(1-4):561–73. doi: 10.1016/j.aquaculture.2003.12.007

[46] Chen SL Li J Deng SP Tian YS Wang QY Zhuang ZM. Isolation of Female-Specific AFLP Markers and Molecular Identification of Genetic Sex in Half-Smooth Tongue Sole (*Cynoglossus Semilaevis*). Mar Biotechnol (2007) 9(2):273–80. doi: 10.1007/s10126-006-6081-x 17308998

[47] Ma KY Chen J Liu ZQ Qiu GF. Inhibitory Effects of RNAi-Mediated Knockdown of EsDmrt-Like Gene on Testicular Development in the Chinese Mitten Crab *Eriocheir Sinensis* . Aquaculture (2016) 463:217–23. doi: 10.1016/j.aquaculture.2016.06.003

[48] Nunes FMF Aleixo AC Barchuk AR Bomtorin AD Grozinger CM Simões ZLP. Non-Target Effects of Green Fluorescent Protein (GFP)-Derived Double-Stranded Rna (dsRNA-GFP) Used in Honey Bee RNA Interference (RNAi) Assays. Insects (2013) 4:90–103. doi: 10.3390/insects401009 26466797 PMC4553431

[49] Gao FX Shi Y Duan W Lu WJ Huang W Zhang XJ. A Rapid and Reliable Method for Identifying Genetic Sex in Obscure Pufferfish (*Takifugu Obscurus)* . Aquaculture (2020) 519:734749. doi: 10.1016/j.aquaculture.2019.734749

[50] Kamiya T Kai W Tasumi S Oka A Matsunaga T Mizuno N. A Trans-Species Missense SNP in *Amhr*2 Is Associated With Sex Determination in the Tiger Pufferfish, *Takifugu Rubripes* (Fugu). PloS Genet (2012) 8(7):e1002798. doi: 10.1371/journal.pgen.1002798 22807687 PMC3395601

[51] Kikuchi K Kai W Hosokawa A Mizuno N Suetake H Asahina K. The Sex-Determining Locus in the Tiger Pufferfish, *Takifugu Rubripes* . Genetics (2007) 175(4):2039–42. doi: 10.1534/GENETICS.106.069278 PMC1855111 17287528

[52] Chamary JV Hurst LD. Evidence for Selection on Synonymous Mutations Affecting Stability of mRNA Secondary Structure in Mammals. Genome Biol (2005) 6(9):1–12. doi: 10.1186/gb-2005-6-9-r75 PMC1242210 16168082

[53] Sauna ZE Kimchi-Sarfaty C. Understanding the Contribution of Synonymous Mutations to Human Disease. Nat Rev Genet (2011) 12(10):683–91. doi: 10.1038/nrg3051 21878961

[54] Liu SF Li LY Fu JJ Liu G Xing XW Lu GX. Molecular Cloning of SRG2, A Mouse Testis Spermatocyte Apoptosis-Related Gene. Acta Biochim Biophys Sin (2002) 34(6):796–9. doi: 10.1007/BF02943277 12417927

[55] Matson CK Murphy MW Sarver AL Griswold MD Bardwell VJ Zarkower D. DMRT1 Prevents Female Reprogramming in the Postnatal Mammalian Testis. Nature (2011) 476(7358):101–4. doi: 10.1038/nature10239 PMC3150961 21775990

[56] Barrionuevo F Scherer G. SOX E Genes: SOX9 and SOX8 in Mammalian Testis Development. Int J Biochem Cell B (2010) 42.3:433–6. doi: 10.1016/j.biocel.2009.07.015 19647095

[57] Han YL Wei JL Cui ZP Zhang J Zhang WJ Wen B. Expression and Cellular Localization of Maternal Factor *Boule* in Gonad of Sea Urchin Mesocentrotus Nudus. J Dalian Fish Univ (2021) 36(2):214–21. doi: 10.16535/j.cnki.dlhyxb.2020-074

[58] Lai F Singh A King ML. Xenopus *Nanos1* Is Required to Prevent Endoderm Gene Expression and Apoptosis in Primordial Germ Cells. Development (2012) 139(8):1476–86. doi: 10.1242/dev.079608 PMC3308181 22399685

[59] Scott AP Ellis T. Measurement of Fish Steroids in Water—a Review. Gen Comp Endocr (2007) 153:392–400. doi: 10.1016/j.ygcen.2006.11.006 17188270

[60] Bannister R Beresford N Granger DW Pounds NA Rand-Weaver M White R. No Substantial Changes in Estrogen Receptor and Estrogen-Related Receptor Orthologue Gene Transcription in *Marisa Cornuarietis* Exposed to Estrogenic Chemicals. Aquat Toxicol (2013) 140:19–26. doi: 10.1016/j.aquatox.2013.05.002 23747549 PMC3778743

[61] Fodor I Urbán P Scott AP Pirger ZJM Endocrinology C. A Critical Evaluation of Some of the Recent So-Called ‘Evidence’for the Involvement of Vertebrate-Type Sex Steroids in the Reproduction of Mollusks. Mol Cell Endocrinol (2020) 516:110949. doi: 10.1016/j.mce.2020.110949 32687858

