# Supplementary material for: Application of SNP in Genetic Sex Identification and Effect of Estradiol on Gene Expression of Sex-Related Genes in Strongylocentrotus intermedius
Source: Front Endocrinol (Lausanne). 2021 Nov 11;12:756530. doi: 10.3389/fendo.2021.756530 (PMC8632358; doi:10.3389/fendo.2021.756530)
Supplement: Supplementary file 1 [file DataSheet_1.zip › Supplementary Material/Supplementary Figures.docx]

Supplementary Material

## Supplementary Figures


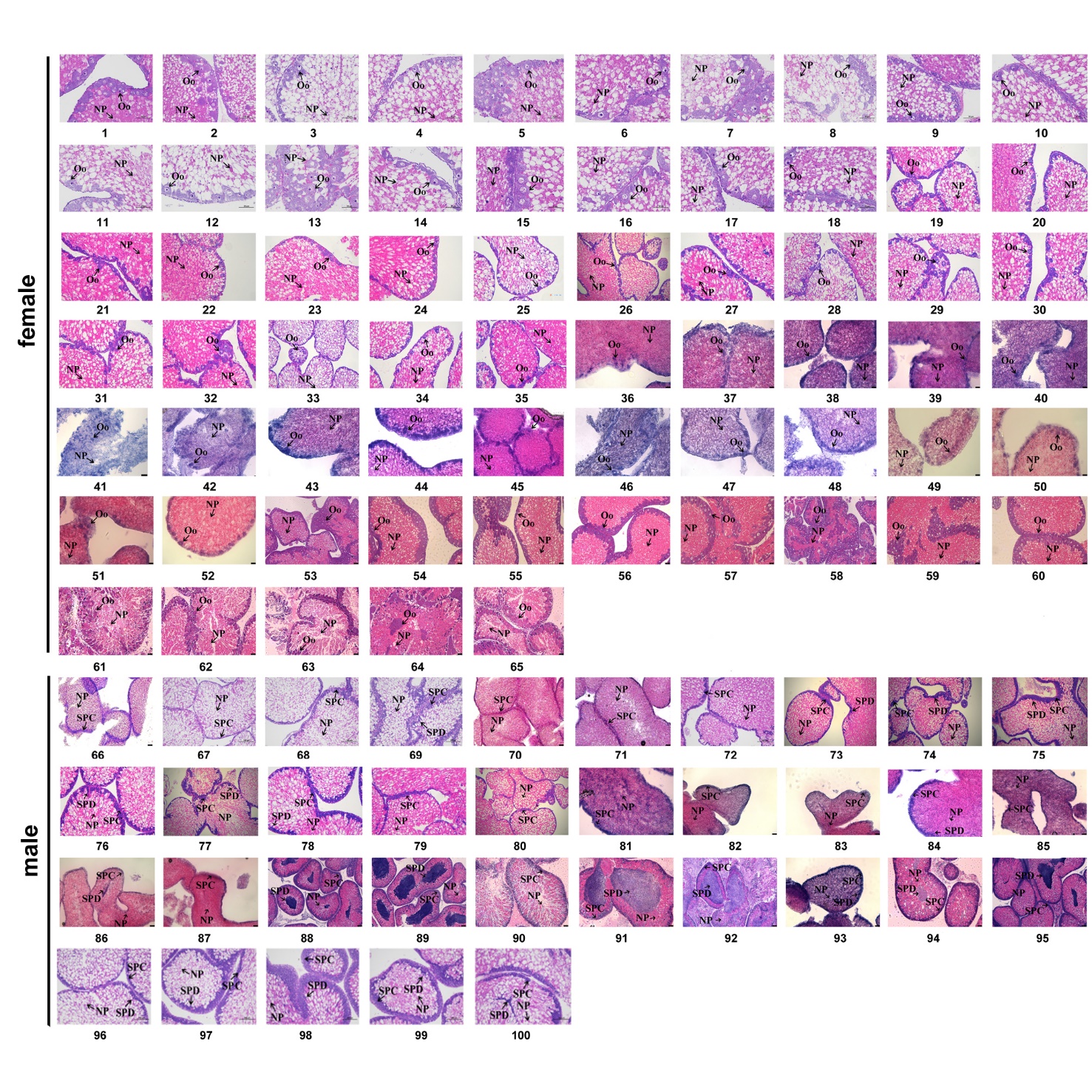


**Supplementary Figure 1.** Sex identification in *S. intermedius* by histological detection of gonads. NP: nutritious phagocytes, SPC: spermatocyte, Oo: oocyte, SPD: spermatid


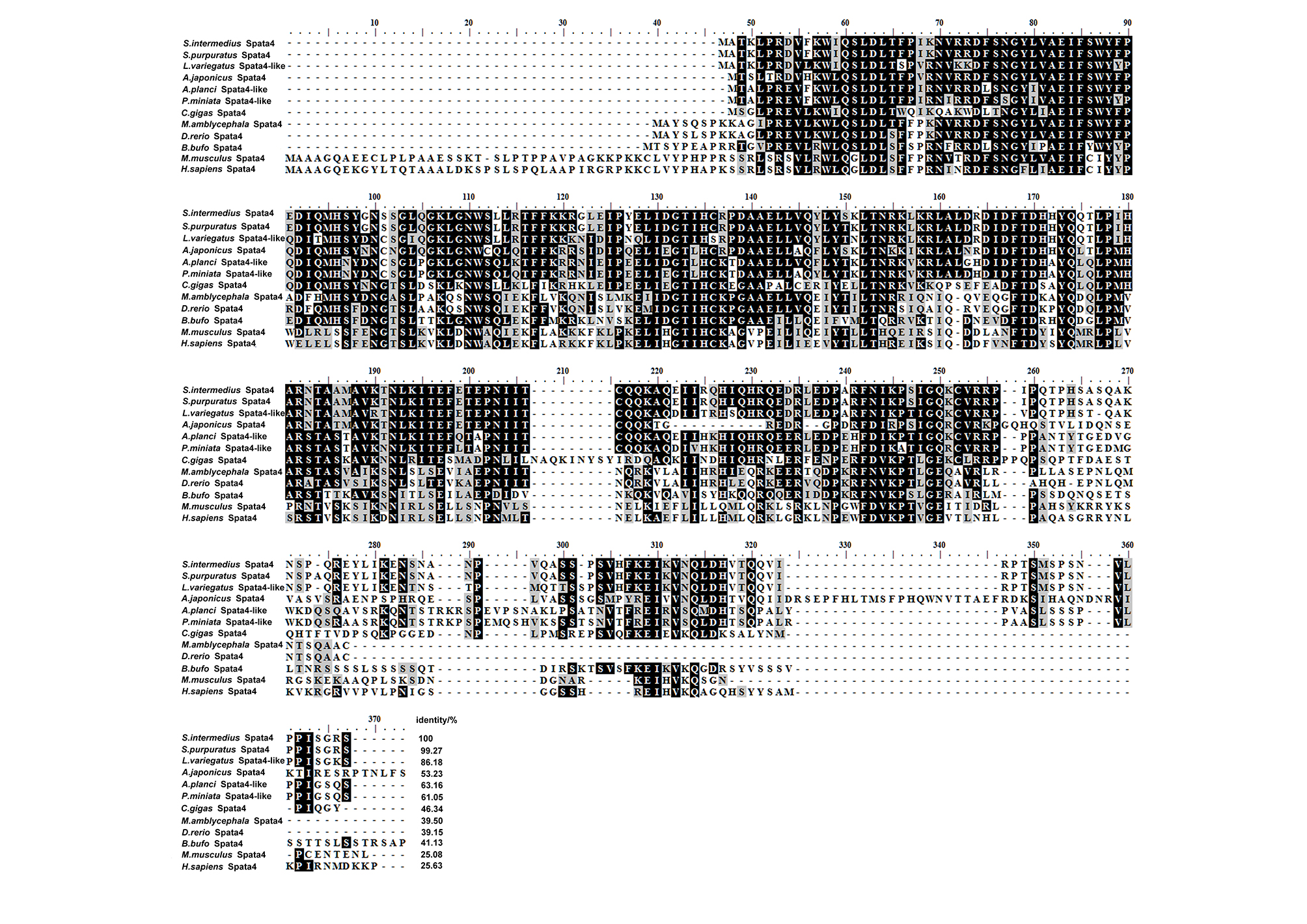


**Supplementary Figure 2.** Multiple protein sequence alignment analysis of Spata4 protein


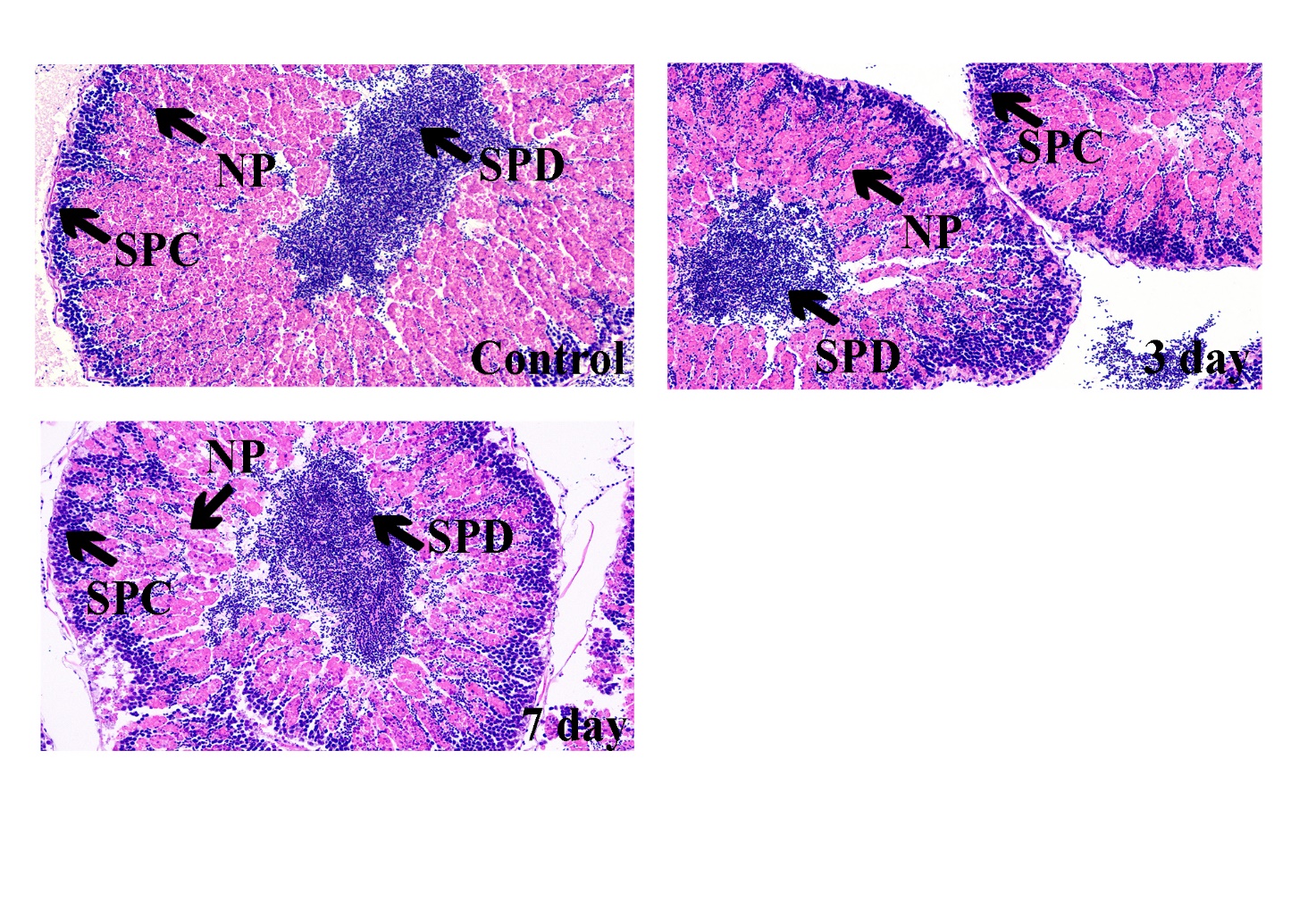
 **Supplementary Figure 3.** Histological detection of testis after RNAi. NP: nutritious phagocytes, SPC: spermatocyte, SPD: spermatid


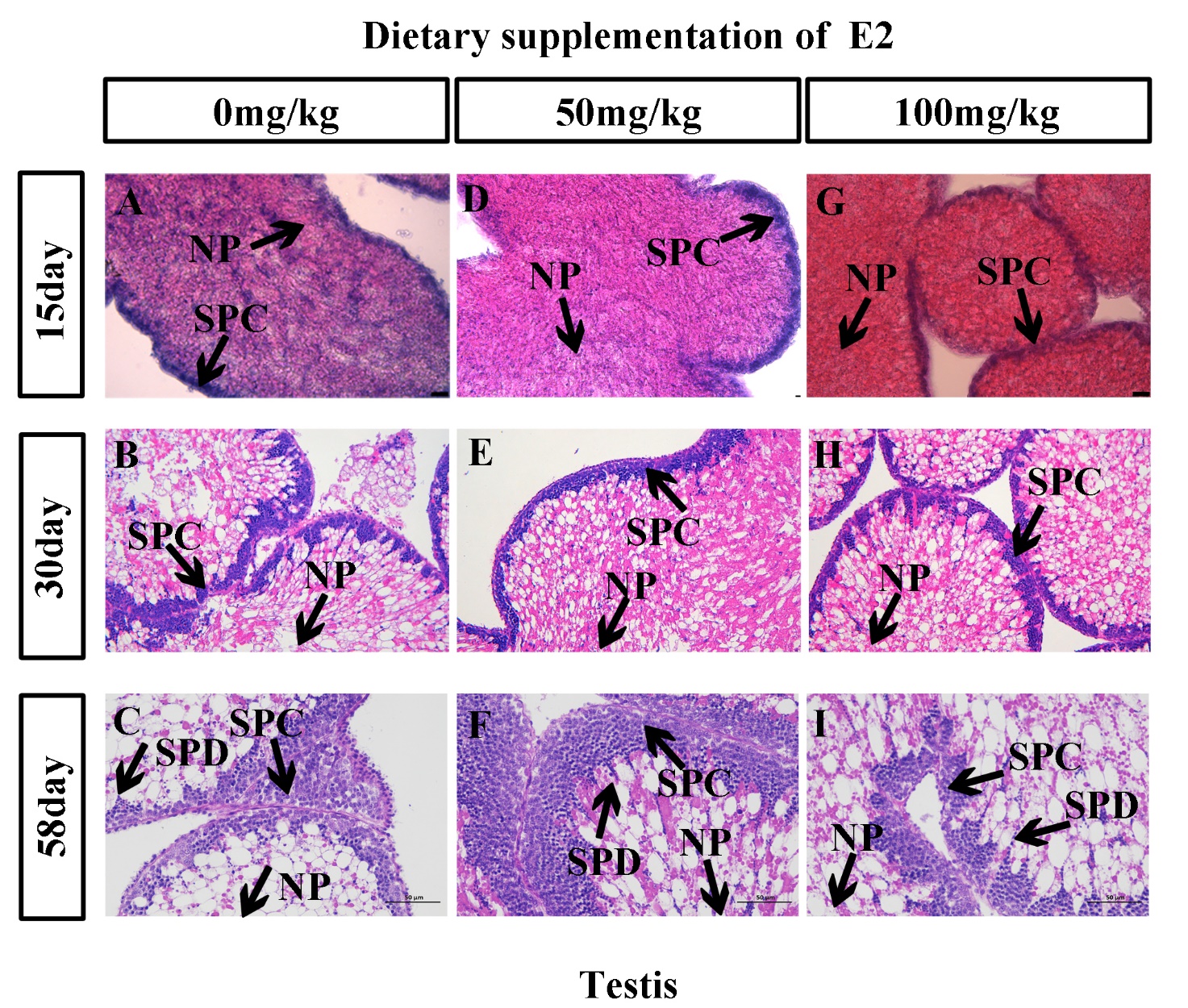


**Supplementary Figure 4.** Histological detection of testis after E2-treated. NP: nutritious phagocytes, SPC: spermatocyte, SPD: spermatid
