# Supplementary material for: Application of SNP in Genetic Sex Identification and Effect of Estradiol on Gene Expression of Sex-Related Genes in Strongylocentrotus intermedius
Source: Front Endocrinol (Lausanne). 2021 Nov 11;12:756530. doi: 10.3389/fendo.2021.756530 (PMC8632358; doi:10.3389/fendo.2021.756530)
Supplement: Supplementary file 1 [file DataSheet_1.zip › Supplementary Material/Table S1 .docx]

Table S1 Primers used in this study

| Primer | Purpose | Sequence (5’-3’) |
| --- | --- | --- |
| female-tag 1F  female-tag 1R | PCR  PCR | GGCCCCACCCCTAAGAAATCCT  TCCCAGCAGGCATTACCCATC |
| female-tag 3F  female-tag 3R | PCR  PCR | TCTTCCATTCCATCAACCAGAC  TACGAGCGGCCCGAAAATGAC |
| female-tag 4F  female-tag 4F | PCR  PCR | GCTTCCTCACACCTCACACCAAATAC  CCGGTCATATATCAAAGACAGAGCC |
| female-tag 6F  female-tag 6R | PCR  PCR | CTACATTGGTAGTGACTTTAGAACTCT  CCTAAGTTATCCATGCAATAGAAAATGATC |
| female-tag 7F  female-tag 7R | PCR  PCR | GCTAGCACATGAGCAGAATGTTAG  GTAATAGTAGTGGCGCTAAAGACG |
| female-tag 8F  female-tag 8R | PCR  PCR | GCATGTCCATATAGATAGTTTATTGTCTG  GTGATGGTTTTGCTCAATAAGCCTATTC |
| female-tag 9F  female-tag 9R | PCR  PCR | GTCCACTGTCATATGTACCCAC  CATGGAGCATCTGTTGCAGGAG |
| female-tag 10F  female-tag 10R | PCR  PCR | GAGGCACTTTTGAGGTACTTG  TCTATCAGAGGCGGACCTC |
| female-tag 11F  female-tag 11R | PCR  PCR | GTGTATTAAACCCTGAAAATTGTACC  CAGTGACATCGAGTGAAGTATC |
| female-tag 12F  female-tag 12R | PCR  PCR | TAGTAGTACACGTACAGGCAAGG  CAAACCTCTCTATAAACGTGGAAAG |
| female-tag 13F  female-tag 13R | PCR  PCR | GCTAGCTCTGCCATACTGTCTCTG  GACAAAAAGGTGGGAGAGACGGTGAG |
| male-tag 1F  male-tag 1R | PCR  PCR | CGTGCATGTCTAGAGAAACCTCATTC  CAAACCTCTCAGCCATTTCCCATCAC |
| male-tag 2F  male-tag 2R | PCR  PCR | GTAGAAGAAGTATCACATTTGGCC  GAGAGAGCCAAAGAAAGCAGGTC |
| male-tag 3F  male-tag 3R | PCR  PCR | CTTGAGTGTCTCCATGACCCTG  CGCTAATGACTTTCACCACTGCC |
| male-tag 4F  male-tag 4R | PCR  PCR | TGGCCCTGGTAACATCAAAATAATC  GTCTAATTTGCCCTGAGAAGCC |
| male-tag 5F  male-tag 5R | PCR  PCR | GACGCATGAAGGATGGCAATC  GGCTAGACTTAGGGTTCCAATCA |
| male-tag 7F  male-tag 7R | PCR  PCR | ATGCCACGAGCAAAGTCCTGAG  TATATCACCAATCTGGGCACATCC |
| male-tag 8F  male-tag 8R | PCR  PCR | CGTGTCTTTCCCGTGGCTTGAATC  GCCTGCAAAGAAAGACCACCTGTC |
| male-tag 9F  male-tag 9R | PCR  PCR | GTTCCCTCGGAGCACAAGTAACA  GCAAGGCATGTGTGTCATTAGCAATC |
| male-tag 10F  male-tag 10R | PCR  PCR | GGGTTCACGTACATCCAAGTAGG  CATTTCATGTATGCCAGTGCGGCC |
| male-tag 11F  male-tag 11R | PCR  PCR | TAACGCCTGGCTGGAATGCTCC  TACCAGTCAGTGCCAAGTGTGCC |
| male-tag 14F  male-tag 14R | PCR  PCR | GCATCTGAGCTGAAGCCGGTGA  GTGGAGCCAGATGTAATCTCAGCG |
| male-tag 15F  male-tag 15R | PCR  PCR | GTATACTCACAAGCCAAGGCTATGATG  TATGACGTCATTGCCAGCTGATTGG |
| male-tag 17F  male-tag 17R | PCR  PCR | GGCCTCAGGTCAACCCTTCATTAG  GACACAGGCAACGACAGAGAGAG |
| male-tag 18F  male-tag 18R | PCR  PCR | TGTCTACGCCACTGTTCTGTTAAG  ATGCACCCTCAATACGCAGGG |
| male-tag 19F  male-tag 19R | PCR  PCR | GCTTGTTCCGAGTATTCTCCG  GACACTTTTTCACTACACGGAC |
| male-tag 20F  male-tag 20R | PCR  PCR | GATTACCAGTTCTGTATGCAGCCAC  CATGGCATTTGATTGGCTGAGAGC |
| male-tag 21F  male-tag 21R | PCR  PCR | GCTGTAGGTCTGTAGTTCATCTCCTG  GACATGGCGACGATTCCACTGCTT |
| male-tag 22F  male-tag 22R | PCR  PCR | CAGCAGCATAAATGATCTTTGATTC  TATCAGAAGTTGGTCTCAACCG |
| male-tag 23F  male-tag 23R | PCR  PCR | GGAATTGATAGTTGATTGCCTGATACAGG  GCAGGAGCAAATGTCGTGTCACC |
| male-tag 24F  male-tag 24R | PCR  PCR | GTCGCTGATGACGACGGGATTATGATG  CGGGCAAAAAGGCAATGCTATCCCAA |
| male-tag 25F  male-tag 25R | PCR  PCR | GTTCTGTGACCTTCGCGACCTCT  GGATTACGATTGATGGGAAAGGAGTAC |
| male-tag 26F  male-tag 26R | PCR  PCR | GTCTATCCAGGAACTTGGGAGAG  CAAATGATGCCAACACAGTTAGCTG |
| male-tag 27F  male-tag 27R | PCR  PCR | TCCACCTCTGTTCTGCCGGAAG  CGGCGCCAATGGACTGATTGCT |
| male-tag 28F  male-tag 28R | PCR  PCR | GGGTGATTGATTGCTTGACTAATGCATTG  CTAGCTTGGAAACTCTTTGCTTCTGTG |
| male-tag 29F  male-tag 29R | PCR  PCR | GGCCTGTAGCACTAAGGTAGTGGT  CGGCGCAACTCGTAA AGTCTAGC |
| male-tag 30F  male-tag 30R | PCR  PCR | GTGTGATGTCTTTTCTGTTTTCGAACGG  CGAGAGGGAGGAGAAGTAGAAGGA |
| male-tag 31F  male-tag 31R | PCR  PCR | GACTCAGTGTTTTGAAGTGATCGCATG  TGGATTCAAGTCAGGGAGGGGTC |
| male-tag 32F  male-tag 32R | PCR  PCR | CTTGAAGCGGCAGTTTCCTTGGAAG  CGTCCGTGTTCCACTGAGACTC |
| male-tag 33F  male-tag 33R | PCR  PCR | GTATAGTCTGGAGAGAGGAGGCC  GGGTTAGGTATAGTGCATGTCAGGTC |
| male-tag 34F  male-tag 34R | PCR  PCR | ATAGTCTGGAGAGAGGAGGCCAATAT  GGAACTAATTGTCGCAGTATGTGTCATAC |
| male-tag 35F  male-tag 35R | PCR  PCR | GTCATCATAAGGAGGAGAAGTCGAAAG  CACCAGGCATGTCACGATTTGATTTC |
| male-tag 36F  male-tag 36R | PCR  PCR | GCTTCCCCTCTCTAGCTCTCTG  TATAATGAGGAGACTCCGTGAAAGCAG |
| male-tag 37F  male-tag 37R | PCR  PCR | TTCTCACAGTACTTTTAAGGTACCG  CAACAAAAAATATGCAGGAGCAGC |
| male-tag 38F  male-tag 38R | PCR  PCR | GACATTCATTCACTATGCCCTGTGTG  GAGATGGAGAGGAGGGGATGAC |
| SNP 1F  SNP 1R | PCR  PCR | GTGAGTTTTGAGGGATCTAGGTTTGGATAG  CCTGACTAGTACATCCCATCGCTTGC |
| SNP 2F  SNP 2R | PCR  PCR | GAGTTGTGTGGGTAGTGAGGATTGAG  CTGATCAATGCACAGGTCTTCAAGGATTC |
| SNP 4F  SNP 4R | PCR  PCR | GTACCTCGGAAACTCCACCCTGTC  GTGCGTGTACGTGTGCGTGACTG |
| SNP 5F  SNP 5R | PCR  PCR | GTGGACTGTTCTTAGCCTGAGAGG  CTGTCCATGGAGGCTTCTGCTATACAT |
| SNP 6F  SNP 6R | PCR  PCR | GGCGAATCCACAGTTAACAAAATGG  CCCATGTTCATTTTTGAATTTTCCC |
| SNP 7F  SNP 7R | PCR  PCR | GATACACAAACACACACGCACCCG  GGCAAGTCCGAGGCAATGTTCCAC |
| SNP 8F  SNP 8R | PCR  PCR | CAAGCAGAATCCATTCCAGTC  GACTGGAATGGATTCTGCTTG |
| SNP 10F  SNP 10R | PCR  PCR | GGTCAGCTTGCTTGCTCTGCCAT  CTCTCTCTAGCTCTCTCTTCATCTAC |
| *spata4*-5’RACE R1 | RACE | CAACAAGGTAGCCATT |
| *spata4*-5’RACE R2 | RACE | CTTCTGACGTTTTTGATAGG |
| *spata4*-5’RACE R3 | RACE | TGAGATCCAAACTTTGAATCC |
| *spata4*-3’RACE F1 | RACE | GCCTCGTCACCCAGCGTTCACTTC |
| *spata4*-3’RACE F2 | RACE | TCAGACCCACCAGCATGTCACCGA |
| *spata4-*F2 | RT-qPCR | CGGCTAGCTTTAGACCGTGATATTG |
| *spata4-*R2 | RT-qPCR | CTTGATGTTGAATCGTGCAGGGTC |
| *spata4*-dsRNA-F | RNAi | TAATACGACTCACTATAGGGCCAATGGCTACCTTGTTGCT |
| *spata4*-dsRNA-R | RNAi | TAATACGACTCACTATAGGGGGTTTGTTTTGACCGCCATA |
| *dmrt1-*F | RT-qPCR | GTTTCTCTCAGTGCCAATCGCACC |
| *dmrt1-*F | RT-qPCR | CGAACGGTCCTGTCTTGTAATTTGCT |
| *soxE-*F | RT-qPCR | AACCTGAAGGAGAGGACCGAGAAG |
| *soxE-*R | RT-qPCR | GAAAACTCCTCCATGCCCATGATGTC |
| *foxl2-*F | RT-qPCR | CGTTTCGGAGCCATCAATGTTGTC |
| *foxl2-*R | RT-qPCR | GACGTAAGAAAATGGCGGCTTCTG |
| *hsd17b8-*F | RT-qPCR | CGGCCGGAATCACTAGAGATCAG |
| *hsd17b8-*R | RT-qPCR | TGACCGATGTTGCCCATAGTGGC |
| *nanos1-*F | RT-qPCR | ATGCCTTTGCAGTTGCCGCTGAAC |
| *nanos1-*R | RT-qPCR | CAGAGTGGACATGTGTAGGCTCG |
| *boule-*F | RT-qPCR | CGATAGCTGCACATCATCATCATCAG |
| *boule-*R | RT-qPCR | GAGACCGGTAGCATACTGCCATTC |

Note: Red fonts indicates the T7 promoter sequence.
