# Supplementary material for: Application of SNP in Genetic Sex Identification and Effect of Estradiol on Gene Expression of Sex-Related Genes in Strongylocentrotus intermedius
Source: Front Endocrinol (Lausanne). 2021 Nov 11;12:756530. doi: 10.3389/fendo.2021.756530 (PMC8632358; doi:10.3389/fendo.2021.756530)
Supplement: Supplementary file 1 [file DataSheet_1.zip › Supplementary Material/Table S2 .docx]

Table S2 GenBank accession numbers for *Spata4* proteins used in alignment and phylogenetic analysis

| Species | GenBank accession number |
| --- | --- |
| *Strongylocentrotus purpuratus* | XP_030837046.1 |
| *Lytechinus variegatus* | XP_041463054.1 |
| *Apostichopus japonicus* | PIK60531.1 |
| *Acanthaster planci* | XP_022109395.1 |
| *Patiria miniata* | XP_038063371.1 |
| *Crassostrea gigas* | XP_034302173.1 |
| *Megalobrama amblycephala* | AFX00809.1 |
| *Danio rerio* | NP_001004013.1 |
| *Bufo bufo* | XP_040274623.1 |
| *Mus musculus* | NP_598472.2 |
| *Homo sapiens* | NP_653245.2 |
