# Supplementary material for: Application of SNP in Genetic Sex Identification and Effect of Estradiol on Gene Expression of Sex-Related Genes in Strongylocentrotus intermedius
Source: Front Endocrinol (Lausanne). 2021 Nov 11;12:756530. doi: 10.3389/fendo.2021.756530 (PMC8632358; doi:10.3389/fendo.2021.756530)
Supplement: Supplementary file 1 [file DataSheet_1.zip › Supplementary Material/Table S3 .docx]

Table S3 Information of candidate sex-specific tags

| Tag name | Ref ID | Sequence |
| --- | --- | --- |
| female-tag 1 | ref19586-1 | ACACTAACTACCATTCCTCCCCCCTTT |
| female-tag 2 | ref19661-1 | ACACTCCCAACAAGATCTCCTAAATTT |
| female-tag 3 | ref44824-1 | ATCAACCAGACTCTGCCTCCACAAACA |
| female-tag 4 | ref50388-1 | ATGGATTCCACTCCTCCTCCTATCCGC |
| female-tag 5 | ref52158-1 | CTTCACCCCACTCCCTCCCCGACATG |
| female-tag 6 | ref70408-1 | CATGTAGTTACCAAAACTCCAATTAAT |
| female-tag 7 | ref86471-1 | CGCCCACGCACTGCCACTCCTGTCGCT |
| female-tag 8 | ref106697-1 | GACAAAATAACATGATCTCCAGACTTG |
| female-tag 9 | ref119950-1 | GCTCCTGCAACAGATGCTCCATGAAAA |
| female-tag 10 | ref136514-1 | GTGTGGGGCACTGTGTCTCCTGCCACC |
| female-tag 11 | ref160312-1 | TCCTCCAAAACAGTATCTCCACCGTGA |
| female-tag 12 | ref173077-1 | TGCCTTGCCACATGTTCTCCGCGCTTT |
| female-tag 13 | ref184143-1 | TTATTTCTCACCGTCTCTCCCACCTTT |
| male-tag 1 | ref4260-1 | AAAGTCAAGACAAGCACTCCATGAGAT |
| male-tag 2 | ref17301-1 | AATTTTATGACTCTCTCTCCTTCTTAC |
| male-tag 3 | ref17526-1 | ACAAAATAAACTAAAACTCCCCGCCGT |
| male-tag 4 | ref26480-1 | ACGTGTCTCACAAGGTCTCCTTCTATT |
| male-tag 5 | ref27507-1 | ACTATTGTGACTTCAACTCCGAACCGA |
| male-tag 6 | ref28524-1 | ATTGAACAAAAACATTATGCAATGTTT |
| male-tag 7 | ref41200-1 | ATAAGAGCTACATTATCTCCCTGTTAT |
| male-tag 8 | ref50499-1 | ATGGCGTAAACCGGTACTCCGTCAGGC |
| male-tag 9 | ref55204-1 | ATTTAAACTACATGTACTCCTGTGCAA |
| male-tag 10 | ref64884-1 | CACTGTTCTACACCCTCTCCTTGTTTG |
| male-tag 11 | ref65902-1 | CAGATTGTGACCAATTCTCCATAACAC |
| male-tag 12 | ref76399-1 | CCCATTGCTACATGCACTCCTTCTATT |
| male-tag 13 | ref77429-1 | CCCCCTTCAACCGTCACTCCACTCGTC |
| male-tag 14 | ref80803-1 | CCGTGTTCCACTGAGACTCCGTCTGGC |
| male-tag 15 | ref82782-1 | GATATATCCACAGCTCTTTCACATATA |
| male-tag 16 | ref84191-1 | CCTTGAGACACAGGTACTCCAAACTAG |
| male-tag 17 | ref89660-1 | CTAAATCTCACTTCTCCTCCTCATTCC |
| male-tag 18 | ref96653-1 | CTCTTTTTAACTTGCCCTCCCCCTATT |
| male-tag 19 | ref100735-1 | CTTCTATTAACTCCCCCTCCATCTCGA |
| male-tag 20 | ref113232-1 | GATTGCACAACACCACCTCCTTTAATC |
| male-tag 21 | ref118202-1 | GAGCAATAGTTCATTGCGCTTACATT |
| male-tag 22 | ref121331-1 | GCTTCTCCAACGGAATCTCCTCGTCGA |
| male-tag 23 | ref122607-1 | GGACCACCTACATCATCTCCTTGGCAA |
| male-tag 24 | ref130713-1 | GTACAAGCTACCCCCCCTCCCCCATGC |
| male-tag 25 | ref133504-1 | GTCCGGAAAACTATAGCTCCTTATGTA |
| male-tag 26 | ref136297-1 | GTGTATGTTACTTGTGCTCCGAGGGAA |
| male-tag 27 | ref139634-1 | GTTTTTGGTACATGTTCTCCGTTTATAG |
| male-tag 28 | ref142271-1 | TAAGTGCCCACCGTCCCTCCGTTAGAG |
| male-tag 29 | ref147784-1 | TAGGAAACTACATTTACTTCATACTCA |
| male-tag 30 | ref160222-1 | TCCTCAACAACATCTCCTCCCTCCTCC |
| male-tag 31 | ref161845-1 | TCGCCATACACCCAACCTCCTCCGAAG |
| male-tag 32 | ref165946-1 | TCTGGCTCCACTAGACCTCCTGAATAT |
| male-tag 33 | ref166402-1 | TCTTACATAACACCCCCTCCTTCTCTT |
| male-tag 34 | ref174271-1 | TGGAATGTTACCTTCACTCCAAGAAAA |
| male-tag 35 | ref189987-1 | TTGATTTGGACACTCGCTCCCTTCATA |
| male-tag 36 | ref191746-1 | TTGTCCCATACCCCACCTCCGCTCCCA |
| male-tag 37 | ref194100-1 | TTTCAATTTACAAGAGCACCTTTGGAG |
| male-tag 38 | ref194909-1 | TTTCCTTTTACTTTCCCTCCTTCCCCA |
