# Supplementary material for: Application of SNP in Genetic Sex Identification and Effect of Estradiol on Gene Expression of Sex-Related Genes in Strongylocentrotus intermedius
Source: Front Endocrinol (Lausanne). 2021 Nov 11;12:756530. doi: 10.3389/fendo.2021.756530 (PMC8632358; doi:10.3389/fendo.2021.756530)
Supplement: Supplementary file 1 [file DataSheet_1.zip › Supplementary Material/Table S4 .docx]

| SNP name | Ref ID | SNP position | Reference Base | Alternative Base | Female | Male |
| --- | --- | --- | --- | --- | --- | --- |
| 1 | ref136785 | 23 | A | T | A/T | A/A |
| 2 | ref38349 | 13 | G | A | G/A | G/G |
| 3 | ref38349 | 26 | G | A | G/A | G/G |
| 4 | ref81593 | 27 | T | A | T/A | T/T |
| 5 | ref128486 | 26 | G | A | G/A | G/G |
| 6 | ref50454 | 9 | G | A | G/A | A/A |
| 7 | ref105876 | 21 | C | A | C/A | C/C |
| 8 | ref119266 | 5 | T | G | T/G | T/T |
| 9 | ref119266 | 14 | C | T | C/T | C/C |
| 10 | ref47585 | 6 | G | A | G/A | G/G |

Table S4 Information of top 10 candidate sex-specific SNPs
